# The S-nitrosylation of parkin attenuated the ubiquitination of divalent metal transporter 1 in MPP^+^-treated SH-SY5Y cells

**DOI:** 10.1038/s41598-020-72630-2

**Published:** 2020-09-23

**Authors:** Yanmin Zhong, Xin Li, Xixun Du, Mingxia Bi, Fengtong Ma, Junxia Xie, Hong Jiang

**Affiliations:** 1 State Key Disciplines: Physiology, Department of Physiology, Shandong Key Laboratory of Pathogenesis and Prevention of Neurological Disorders, School of Basic Medicine, Qingdao University, Qingdao, 266071 China; 2 Office of Drug Clinical Trial Management, The Affiliated Hospital of Qingdao University, Qingdao, 266003 China; 3 Clinic Medicine, Medical College, Qingdao University, Qingdao, 266071 China

**Keywords:** Neuroscience, Diseases

## Abstract

Abnormal iron accumulation caused by elevated levels of divalent metal transporter 1 (DMT1) contributes to progressive neurodegeneration in Parkinson's disease (PD). Parkin is a E3 ubiquitin ligase for the ubiquitination of DMT1. S-nitrosylated parkin (SNO-parkin) is commonly observed in PD. However, the effects of S-nitrosylation on the E3 ubiquitin ligase activity of parkin for the ubiquitination of DMT1 in PD are largely unknown. To elucidate the role of S-nitrosylated parkin and DMT1 in PD, SH-SY5Y cells were transfected with parkin, being treated with S-nitrosoglutathione (GSNO) and 1-methyl-4-phenylpyridinium (MPP^+^). The results showed increased levels of oxidized nitric oxide (NO) and S-nitrosylated parkin after the treatment of GSNO and MPP^+^ in parkin-transfected cells. Consistently, increased levels of DMT1, iron uptake and cell viability were observed. Interestingly, inhibition of S-nitrosylated parkin reduced the level of DMT1. Further, S-nitrosylation of parkin significantly inhibited the ubiquitination of DMT1. When HEK293T cells were transfected with plasmid of parkin with single site mutation (Cys241A, Cys260A, Cys323A), ubiquitination of DMT1 was also inhibited. However, the cells cotransfected with plasmids containing all three mutations, GSNO treatment did not affect the ubiquitination of DMT1. The expression of SNO-parkin and DMT1 protein in substantia nigra increased significantly gradually after 2 h, 4 h and 24 h with MPTP injection. These results indicate that the S-nitrosylation of parkin inhibits its E3 ubiquitin ligase activity for the ubiquitination of DMT1, which contributes to iron accumulation and degenerative process in PD. Targeted S-nitrosylation could provide a potential therapeutic strategy against PD.

## Introduction

Parkinson's disease (PD) is a common neurodegenerative disease involving the loss of dopaminergic neurons in the substantia nigra^1,2^. The underlying mechanisms leading to neuronal degeneration in PD are largely unknown. Both genetic and environmental factors are involved. Iron plays an important role in nigral dopaminergic neuronal degeneration in PD^3–8^. Our previous studies showed that divalent metal transporter 1 (DMT1), the first identified iron import protein in mammals, was upregulated in PD animal models^4,9,10^. The transcriptional regulation of DMT1 relied on iron responsive protein (IRP) and iron regulatory element (IRE)^11^.

PARK2, cloned in 1998, is a causative gene for PD, whose mutation leads to autosomal recessive juvenile PD^12,13^. The major post-translational modifications of parkin, including phosphorylation, ubiquitination, acetylation, and nitration, can modify the activity of parkin as an E3 ubiquitin ligase^14^. Parkin is responsible for the ubiquitination of a variety of substrate including DMT1^15^. S-nitrosylation, another post-translational modification, involves the covalent attachment of nitric oxide (NO) to a cysteine thiol group of the target protein^16^. Although the S-nitrosylation of parkin is commonly observed in PD^17–19^, the effects of S-nitrosylation on the E3 ubiquitin ligase activity of parkin for the ubiquitination of DMT1 in PD are largely unknown.

The elevated production of reactive nitrogen species caused by nitrosative stress is damaged to dopaminergic neurons in substantia nigra during PD^16^. The S-nitrosylation of parkin was also observed in postmortem human brains with PD^17^. The role of S-nitrosylated parkin (SNO-parkin) in PD was investigated in the present study. S-nitrosoglutathione (GSNO), a NO donor, can cause an increased intracellular NO content, further leading to the S-nitrosylation of parkin^16^. 1-Methyl-4-phenylpyridinium (MPP^+^), as a common agent for generating PD models, inhibits complex I of the mitochondrial respiratory chain to induce neuronal death^20^. Using SH-SY5Y cells under GSNO or MPP^+^ treatment, the interaction between S-nitrosylated parkin and DMT1 was studied. HEK293T cells were also employed to elucidate the underlying mechanisms of ubiquitination of DMT1. This study might provide a new clue about the degradation of DMT1 under nitrosative stress, which implied a potential therapeutic target for PD.

## Results

### Protein levels of DMT1 decreased in parkin-transfected SH-SY5Y cells

**Figure 1:**
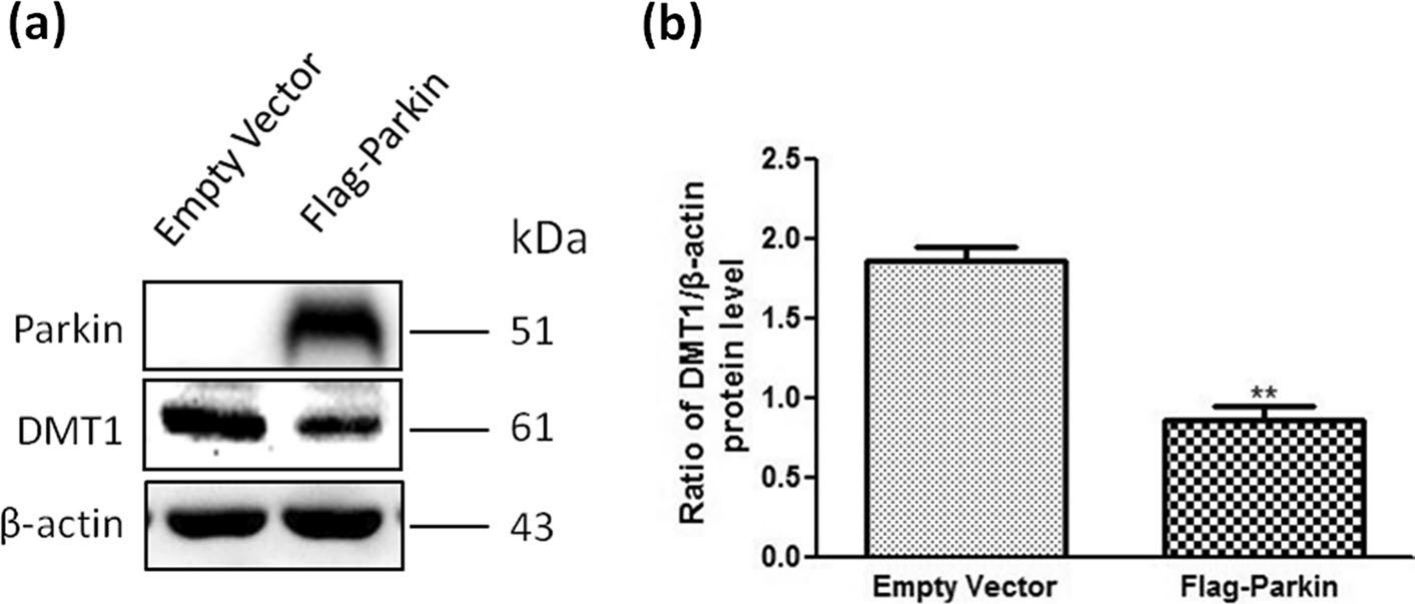
Effect of parkin on DMT1 levels in SH-SY5Y cells. (**a**) Changes of DMT1 levels when parkin were transfected into SH-SY5Y cells for 24 h. (**b**) Statistical analysis. Data were presented as the ratio of DMT1/β-actin. **P = 0.0012, Flag-parkin versus empty vector. N = 9.

SH-SY5Y cells were applied because the endogenous level of parkin expression is too low to be detected. First, we investigated the protein level of DMT1 in SH-SY5Y cells after transfection of parkin. The level of DMT1 decreased by 53.9% compared with that of the control (transfection of empty vector), as shown in Fig. 1 (*P* = 0.012). The results indicated that overexpression of parkin promoted the reduction of DMT1.

### S-nitrosylation of parkin resulted in an increase of DMT1 level in parkin-transfected SH-SY5Y cells

**Figure 2:**
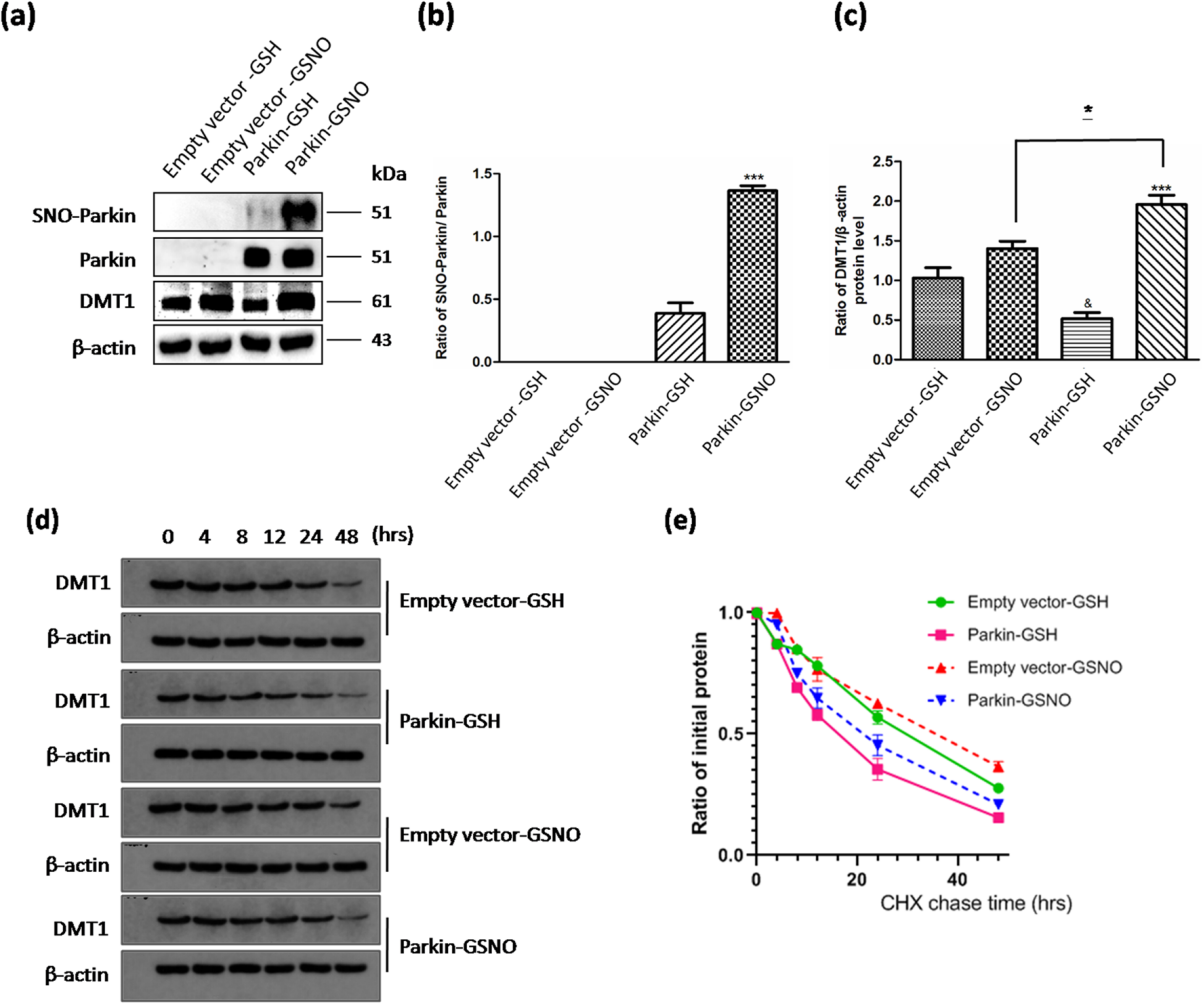
Effect of GSNO on DMT1 levels in parkin-overexpressing SH-SY5Y cells. (**a**) Changes of SNO-parkin and DMT1 protein levels in SH-SY5Y cells transiently expressing parkin with 200 μM of GSNO or GSH treatment for 24 h. (**b**) Statistical analysis of changes in SNO-parkin levels. Data were presented as the ratio of SNO-parkin/parkin. ***P = 0.0002, parkin-GSNO versus parkin-GSH. (**c**) Statistical analysis of changes in DMT1 levels. Data were presented as the ratio of DMT1/β-actin. ^&^*P* = 0.013, parkin-GSH versus empty vector-GSH; ***P = 0.0003, parkin-GSNO versus parkin-GSH; *P = 0.025, parkin-GSNO versus empty vector-GSNO. N = 9. (**d**) DMT1 protein half-life (t1/2) was determined by pulse-chase assay and immunoblotting. CHX was administered in SH-SY5Y cells with different treatment to block protein synthesis. (**e**) Statistical analysis of DMT1 protein half-life (t1/2). *P* = 0.027, parkin-GSH versus empty vector-GSH; *P* = 0.034, parkin-GSNO versus empty vector-GSNO; *P* = 0.121, empty vector-GSNO versus empty vector-GSH; *P* = 0.074, parkin-GSNO versus parkin-GSH. N = 6.

Parkin-transfected SH-SY5Y cells were treated with 200 μM GSNO for 24 h. Under such conditions, parkin was S-nitrosylated in Biotin-Switch Assay (Fig. 2a,b, *P* = 0.0002). Meanwhile, a 2.77-fold significantly increase in DMT1 levels was observed, compared to those in cells with GSH treatment (Fig. 2a,c, *P* = 0.0003). Interestingly, levels of DMT1 elevated in Parkin-GSNO group, compared with Empty vector-GSNO group (Fig. 2a,c , *P* = 0.025). The results indicated that S-nitrosylation of parkin inhibited the expression of DMT1. Confirmly, we still found that transfected parkin downregulated the protein level of DMT1 (Fig. 2a,c, *P* = 0.013).

In order to explore whether GSNO can affect the level of DMT1 directly, pulse chase experiments were executed. The half-life of DMT1 was shortened when parkin transfected (Fig. 2d,e, *P* = 0.027, parkin-GSH vs. empty vector-GSH; *P* = 0.034, parkin-GSNO vs. empty vector-GSNO). However, the half-life of DMT1 was unchanged when GSNO added (Fig. 2d,e, *P* = 0.121, empty vector-GSNO vs. empty vector-GSH; *P* = 0.074, parkin-GSNO vs. parkin-GSH). The results prompt GSNO could not affect the level of DMT1 directly (Supplementary Information 1).

### GSNO and MPP^+^ treatment increased the levels of oxidized NO in parkin-transfected SH-SY5Y cells

**Figure 3:**
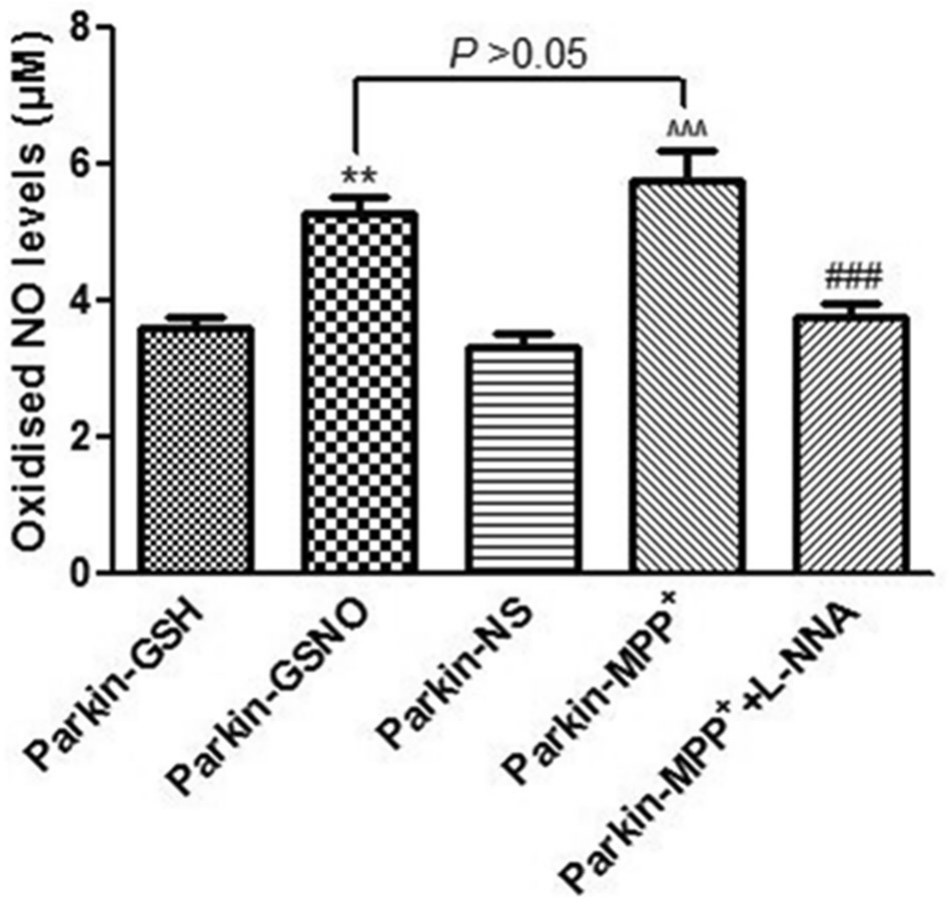
GSNO and MPP^+^ increased NO generation in parkin-transfected SH-SY5Y cells. Changes of oxidized NO levels following different treatment (200 μM of MPP^+^; 200 μM of GSNO; 200 μM of GSNO + 1 mM of L-NNA) for 24 h in parkin-transfected SH-SY5Y cells. **P = 0.004, parkin-GSNO versus parkin-GSH; ^^^^^*P* = 0.0007, parkin-MPP^+^ versus parkin-NS; ^###^*P* = 0.0009, parkin-MPP^+^ + L-NNA versus parkin-MPP^+^. *P* = 0.063, parkin-MPP^+^ versus parkin-GSNO. N = 6.

Excessive levels of NO cause a variety of protein thiol nitrosylations. In order to determine the nitrosylation environment in cells with the treatment of GSNO and MPP^+^, we measured the amount of oxidized NO. Due to the rapid conversion of NO to nitrite (NO_2_) and nitrate (NO_3_), the total amount NO_2_ and NO_3_ was used to represent NO production. The results showed that the oxidized NO (NO_2_ + NO_3_) levels were increased significantly by 46.5% (Fig. 3, *P* = 0.004, parkin-GSNO vs. parkin-GSH) and 60.2% (Fig. 3, *P* = 0.0007, parkin- MPP^+^ vs. parkin-NS) in the GSNO and MPP^+^ treatment groups, respectively. When NOS inhibitor L-NNA was added, the levels of oxidized NO were decreased by 35.1% (Fig. 3, *P* = 0.0009, parkin-MPP^+^ + L-NNA vs. parkin-MPP^+^). These results indicated that both GSNO and MPP^+^ could induce increased levels of oxidized NO, leading to protein thiol nitrosylations.

### MPP^+^ treatment increased the levels of S-nitrosylated parkin and DMT1 in parkin-transfected SH-SY5Y cells

**Figure 4:**
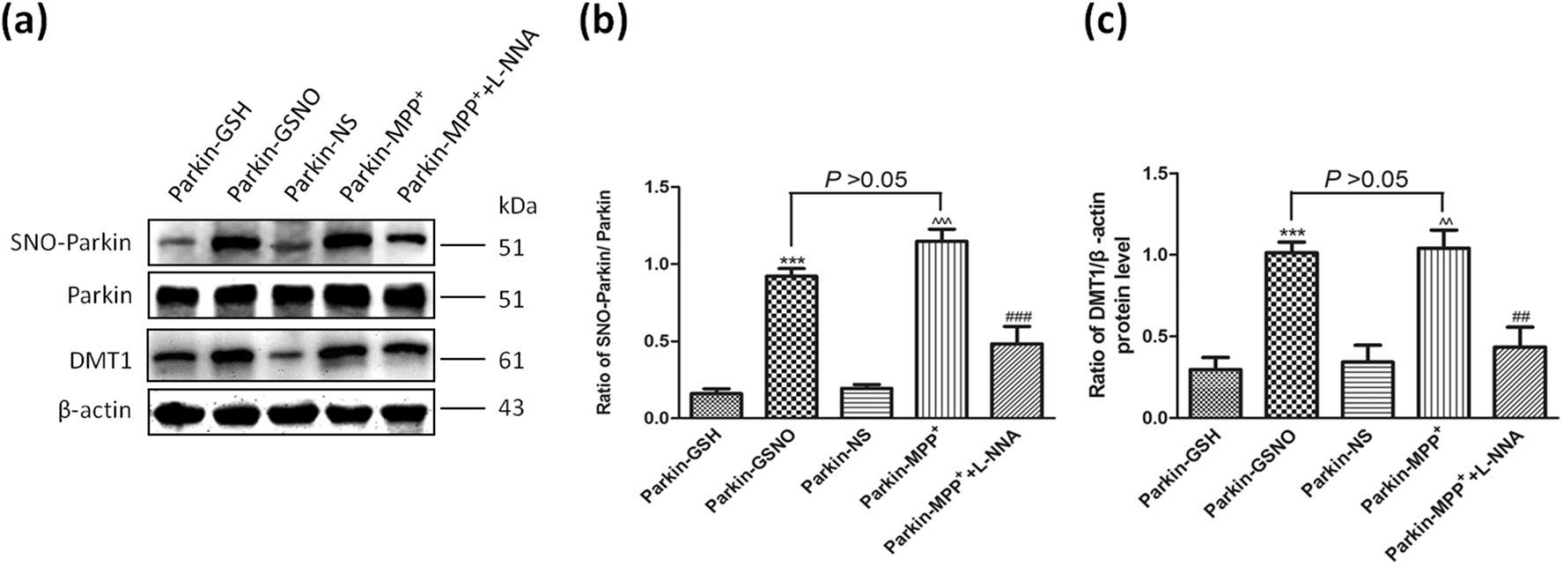
GSNO and MPP^+^ increased the levels of S-nitrosylated parkin and DMT1 in parkin-transfected SH-SY5Y cells. (**a**) Changes of SNO-parkin and DMT1 protein levels following different treatment (200 μM of MPP^+^; 200 μM of GSNO; 200 μM of MPP^+^ + 1 mM of L-NNA) for 24 h in parkin-transfected SH-SY5Y cells. (**b**) Statistical analysis for changes in levels of SNO-parkin. Data were presented as the ratio of SNO-parkin/parkin. ***P = 0.0002, parkin-GSNO versus parkin-GSH; ^^^^^*P* = 0.0002, parkin-MPP^+^ versus parkin-NS; ^###^*P* = 0.0004, parkin-MPP^+^ + L-NNA versus parkin-MPP^+^; *P* = 0.0541, parkin-MPP^+^ versus parkin-GSNO. (**c**) Statistical analysis for changes in levels DMT1. Data were presented as the ratio of DMT1/β-actin. GSNO treatment acted as a positive control for MPP^+^ treatment. ***P = 0.0004, parkin-GSNO versus parkin-GSH; ^^^^*P* = 0.0027, parkin-MPP^+^ versus parkin-NS; ^##^*P* = 0.0081, parkin-MPP^+^ + L-NNA versus parkin-MPP^+^; *P* = 0.847, parkin-MPP^+^ versus parkin-GSNO. N = 9.

**Figure 5:**
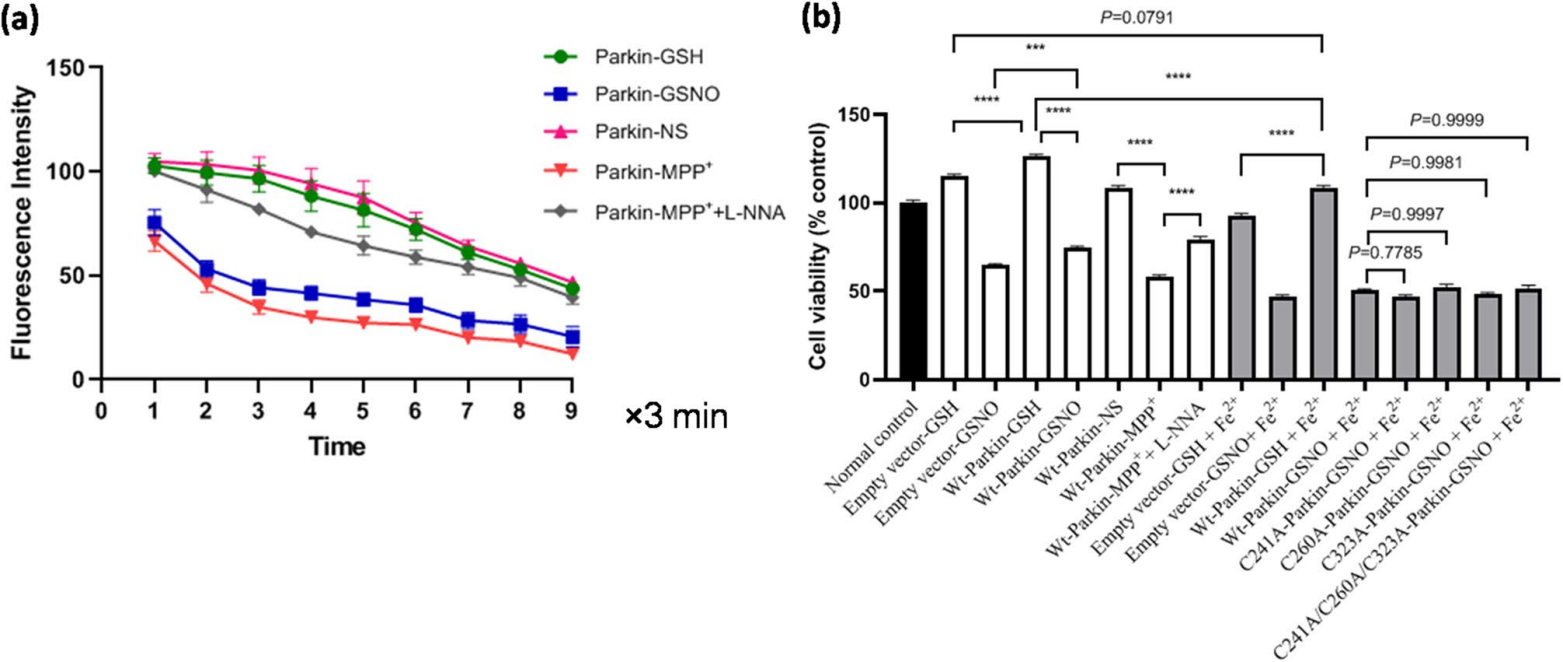
The S-nitrosylation of parkin enhanced ferrous iron influx and reduced cell viability in parkin-transfected SH-SY5Y cells. (**a**) Statistical analysis for changes in ferrous iron uptake, following different treatment (200 μM of MPP^+^; 200 μM of GSNO; 200 μM of MPP^+^ + 1 mM of L-NNA) for 24 h in parkin-transfected SH-SY5Y cells. *P* = 0.001, parkin-GSNO versus parkin-GSH; *P* = 0.0008, parkin-MPP^+^ versus parkin-NS; *P* = 0.0023, parkin-MPP^+^ + L-NNA versus parkin-MPP^+^. N = 6. (**b**) Statistical analysis for cells viability following different treatment for 24 h in parkin-transfected SH-SY5Y cells. ****P = 0.0000, ***P = 0.0004. N = 6.

When parkin-transfected cells were treated with 200 μM MPP^+^ for 24 h, SNO-parkin was increased significantly (Fig. 4a,b, *P* = 0.0002, parkin-MPP^+^ vs. parkin-NS), to the same extent as that following treatment with 200 μM GSNO (Fig. 4a,b, *P* = 0.0541). Meanwhile, the levels of DMT1 were increased significantly by 2.03-fold in MPP^+^-treated SH-SY5Y cells, compared with that of the parkin-NS group (Fig. 4a,c, *P* = 0.0027). When L-NNA was add, the levels of S-nitrosylated parkin and DMT1 recovered (Fig. 5).

### S-nitrosylation of parkin aggravated Fe^2+^-induced reduction of cell viability in parkin-transfected SH-SY5Y cells

In view of the transport role of iron ions by DMT1 in neuron degeneration of PD, we further observed the iron uptake in parkin-transfected SH-SY5Y cells using a laser confocal microscopy. Under the treatment with 200 μM of GSNO or 200 μM of MPP^+^ for 24 h, intensity of fluorescence was significantly reduced (Fig. 5a, *P* = 0.001, parkin-GSNO vs. parkin-GSH; *P* = 0.0008, parkin-MPP^+^ vs. parkin-NS). There were no differences between the parkin-MPP^+^ group and the parkin-GSNO group (Fig. 5a, *P* = 0.087, parkin-MPP^+^ vs. parkin-GSNO). The results indicated that S-nitrosylation of parkin aggravated the iron uptake.

Consistently, we also observed that the cell viability from GSNO treatment group or MPP^+^ was decreased significantly measured CellTiter-Glo Luminescent Cell Viability Assay Kit (Fig. 5b, *P* = 0.0000, Wt-Parkin-GSNO vs. Wt-Parkin-GSH; Wt-Parkin- MPP^+^ vs. Wt-parkin-NS). When L-NNA was added, the iron uptake and cell viability recovered (Fig. 5b).

In order to verify SNO-parkin participate in iron uptake-related cell death further, SH-SY5Y cells were treated with 100 μM FeSO_4_ for 24 h. As shown in Fig. 5b, ferrous treatment could significantly reduce the cell viability in SH-SY5Y cells, compared with that of the control (*P* = 0.0000, Wt-Parkin-GSH + Fe^2+^ vs. Wt-Parkin-GSH). When SH-SY5Y cells were transfected with Wt-parkin, the cell viability increased significantly (*P* = 0.0000, Empty vector-GSH + Fe^2+^ vs. Wt-Parkin-GSH + Fe^2+^; *P* = 0.0000, Empty vector-GSH vs. Wt-Parkin-GSH; *P* = 0.0000, Empty vector-GSNO vs. Wt-Parkin-GSNO; P = 0.0791, Empty vector-GSH vs. Wt-Parkin-GSH + Fe^2+^). The results indicated that S-nitrosylation of parkin aggravates Fe^2+^-induced reduction of cell viability.

According to previous reports on parkin, Cys241, Cys260, and Cys323 are predicted to be parts of the consensus motif of S-nitrosylation^21,22^. To examine whether the mutation of S-nitrosylated parkin had an effect on the cell viability, SH-SY5Y cells were transfected with plasmid of parkin with the site mutations (Cys241A, Cys260A, Cys323A) respectively or collectively. Changes in cell viability were not observed (Fig. 5b, *P* = 0.7785, Wt-Parkin-GSNO + Fe^2+^ vs. C241A-Parkin-GSNO + Fe^2+^; *P* = 0.9997, Wt-Parkin-GSNO + Fe^2+^ vs. C260A-Parkin-GSNO + Fe^2+^; *P* = 0.9981, Wt-Parkin-GSNO + Fe^2+^ vs. C323A-Parkin-GSNO + Fe^2+^; *P* = 0.9999, Wt-Parkin-GSNO + Fe^2+^ vs. C241A/C260A/C323A-Parkin-GSNO + Fe^2+^).

### S-nitrosylation impaired the E3 ubiquitin ligase activity of parkin and inhibited the ubiquitination of DMT1

**Figure 6:**
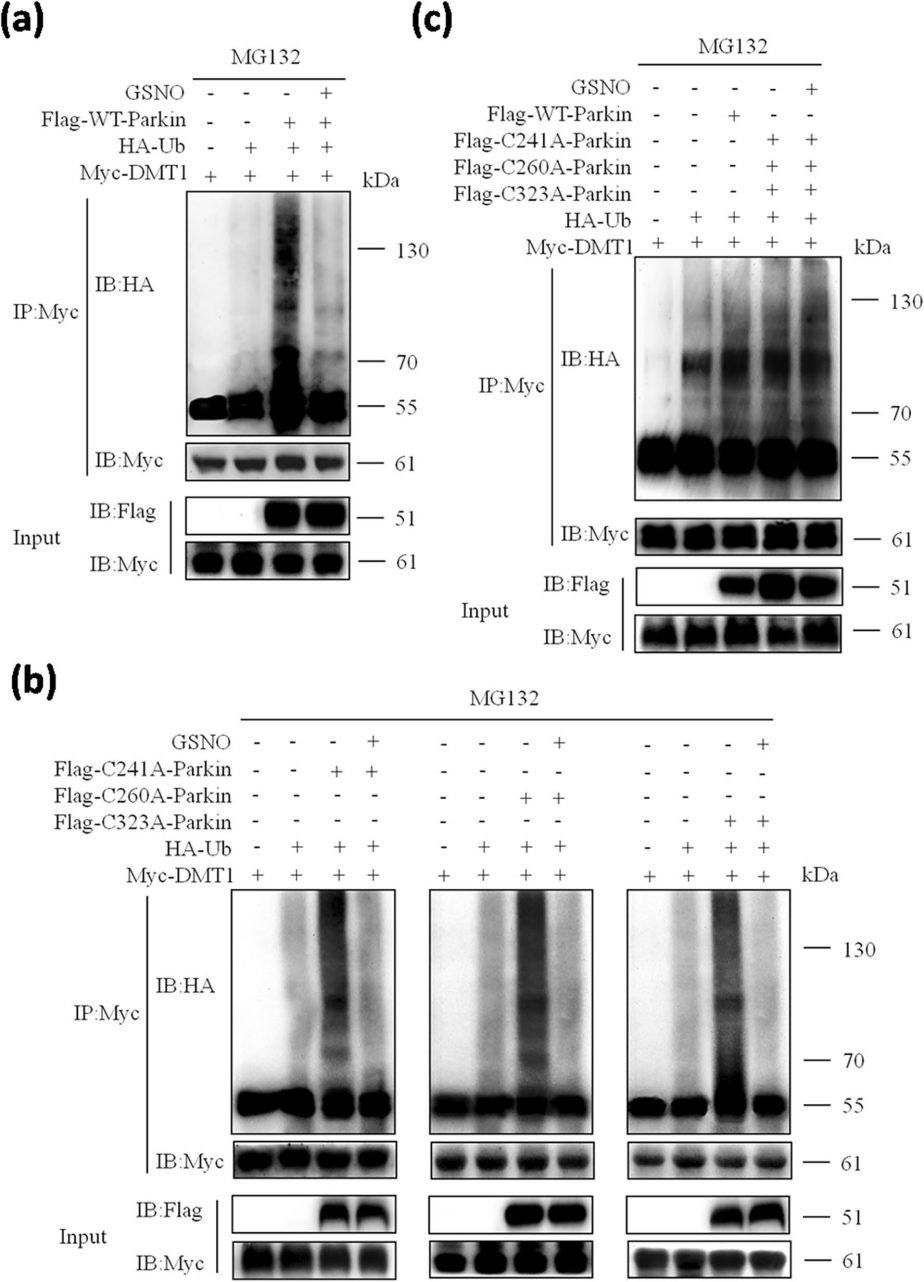
S-nitrosylation impaired the E3 ubiquitin ligase activity of parkin and inhibited the ubiquitination of DMT1. (**a**) WT-parkin was co-transfected with Myc-DMT1 and HA-Ub into HEK293T cells for 24 h. The ubiquitination of DMT1 presented. With the GSNO treatment, the ubiquitination of DMT1 was inhibited. (**b**) Single site mutant (Cys241A, Cys260A, Cys323A)-parkin was co-transfected with Myc-DMT1 and HA-Ub into HEK293T cells for 24 h. With the GSNO treatment, the ubiquitination of DMT1 was inhibited also. (**c**) Mutants (Cys241A, Cys260A, Cys323A)-parkin simultaneously, were co-transfected with Myc-DMT1 and HA-Ub into HEK293T cells for 24 h. With the GSNO treatment, the ubiquitination of DMT1 was not inhibited. Panels were representative of 3 independent experiments.

To further investigate the influence of nitrosative stress on the E3 ubiquitin ligase activity of parkin, we performed intracellular ubiquitination experiments*.* As shown in Fig. 6a, When Flag-parkin joined, immunoprecipitated DMT1 elevated the levels of HA-Ub, which indicated parkin mediated ubiquitination of DMT1. However, the ubiquitination of DMT1was significantly was decreased with GSNO treatment. The results indicate that the S-nitrosylation of parkin inhibits its E3 ubiquitin ligase activity for the ubiquitination of DMT1.

To examine whether the mutation of S-nitrosylated parkin had an effect on its ubiquitination function, HEK293T cells were transfected with plasmid of parkin with the single site mutations (Cys241A, Cys260A, Cys323A, respectively). The results indicated that S-nitrosylation of parkin significantly inhibited the ubiquitination of DMT1 in single site mutant parkin-transfected HEK293T cells (Fig. 6b). However, when cells were cotransfected with all three mutant parkin plasmids, GSNO treatment did not affect the ubiquitination of DMT1 (Fig. 6c). These results prompted problably that residues Cys241, Cys260 and Cys323 worked together to regulate the E3 ubiquitin ligase activity of SNO-parkin.

### The protein levels of SNO-parkin and DMT1 were changed in substantia nigra from PD mice

**Figure 7:**
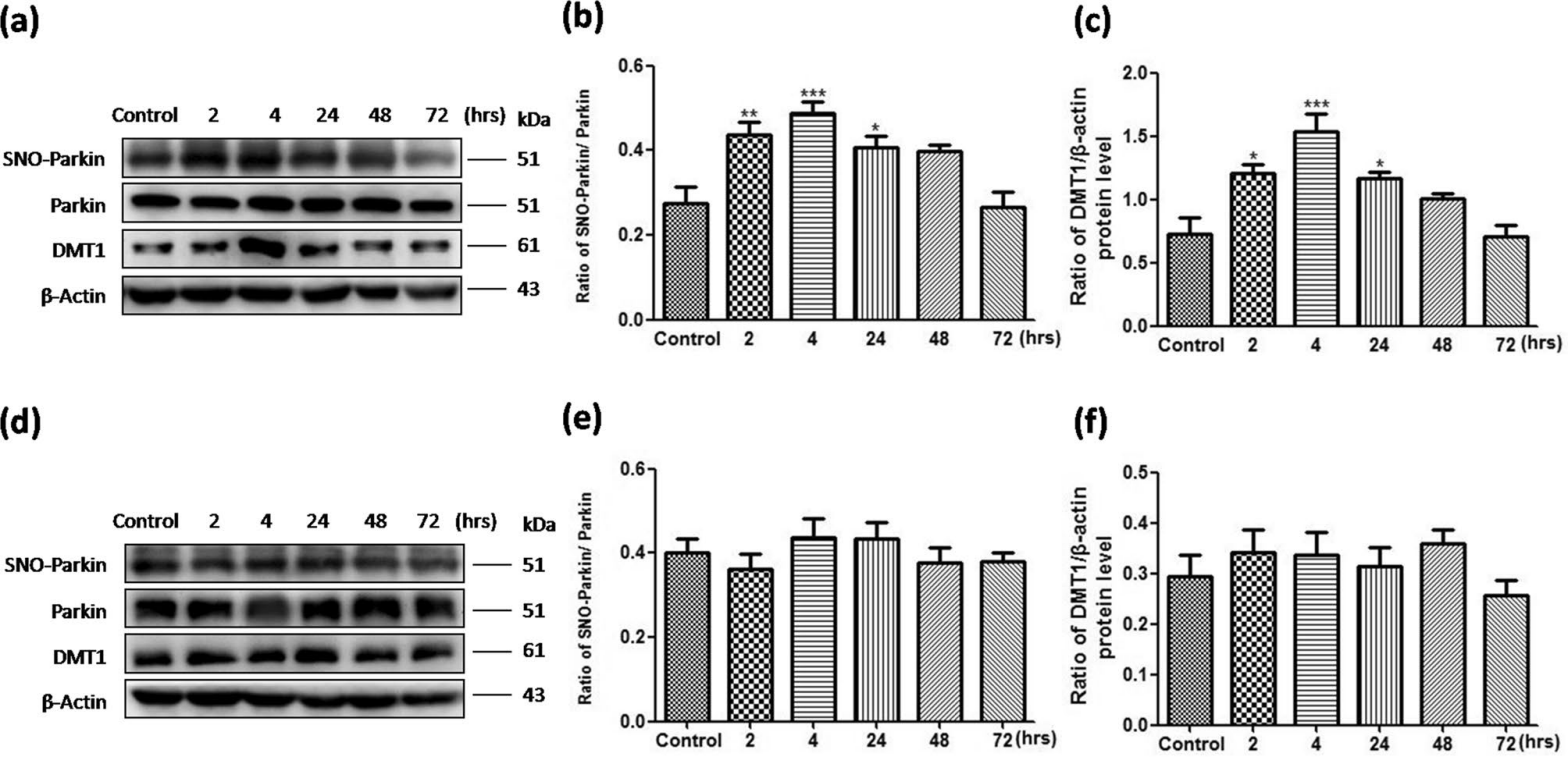
In PD mice, the protein levels of SNO-parkin and DMT1 in substantia nigra were changed. (**a**) The expression of SNO-parkin and DMT1 protein in substantia nigra was detected in MPTP induced PD mice. (**b**) Statistical analysis for changes in levels of SNO-parkin. **P = 0.001, 2 h versus control; ***P = 0.0008, 4 h versus control; *P = 0.03, 24 h versus control. (**c**) Statistical analysis for changes in levels of DMT1. *P = 0.02, 2 h versus control; ***P = 0.0007, 4 h versus control; *P = 0.04, 24 h versus control. (**d**) The expression of SNO-parkin and DMT1 protein in striatum was detected in MPTP induced PD mice. (**e**) Statistical analysis for changes in levels of SNO-parkin. *P* = 0.611. (**f**) Statistical analysis for changes in levels of DMT1. *P* = 0.472. N = 6.

In Fig. 7a–c, Western Blot results showed that the expression of SNO-parkin (**P = 0.001, 2 h vs. control; ***P = 0.0008, 4 h vs. control; *P = 0.03, 24 h vs. control.) and DMT1 protein (*P = 0.02, 2 h vs. control; ***P = 0.0007, 4 h vs. control; *P = 0.04, 24 h vs. control) in substantia nigra increased significantly gradually after 2 h, 4 h and 24 h with MPTP injection. However, No change of SNO-parkin and DMT1 protein was observed in striatum (Fig. 7d–f).

## Discussion

In this study, we observed that nitrosative stress induced by GSNO or MPP^+^ promoted S-nitrosylation of parkin, resulting in the elevated expression of DMT1 and subsequent iron influx related cell death. The Cys241, Cys260 and Cys323 residues in parkin cooperate in the E3 ubiquitin ligase activity by S-nitrosylation. These results demonstrated that S-nitrosylation inhibits the E3 ubiquitin ligase activity of parkin, leading to impaired ubiquitination of DMT1and iron accumulation during the degenerative process in PD.

Abnormal iron accumulation is involved in several neurological disorders. Iron delivery systems provide a requisite supply of iron for mitochondrial respiratory chain complexes and iron-containing proteins^23–27^. Over the last decade, a number of proteins regulated by transcriptional and posttranscriptional processes have been identified that are involved in both iron absorption and homeostatic iron trafficking^28,29^. DMT1 plays an important role in the function of transferrin, which is the major iron transporter that allows iron to enter mammalian cells and to be excreted from endosomes^30^. As reported, the expression of DMT1 is partially regulated by the proteasomal pathway^31,32^. Two different E3 ubiquitin ligases participate in the ubiquitination of DMT1: parkin and WW domain-containing protein 2 (WWP2)^15,33,34^. As an important post-translational modification, ubiquitination regulates cell biological processes in many ways^35^. The attachment of ubiquitin or the polyubiquitin chain to the protein regulates turnover, localization and activity of substrate.

The parkin protein consists of a ubiquitin-like domain in its N-terminus, a unique parkin-specific domain, an IBR domain, RING1 and RING2 domains^36^. Parkin ubiquitinates a variety of cytosolic and outer mitochondrial membrane substrates during mitochondrial depolarization^37^. As reported, parkin is an E3 ubiquitin ligase that is responsible for the ubiquitination of DMT1. Overexpression of parkin in SH-SY5Y cells resulted in a reduction of the 1B-DMT1 isoform and manganese transport^15^. In addition, a few of substrates were found to be accumulated in parkin deficient mice brain or in disease phenotypes. P62, a critical regulator for protein quality control, inclusion body formation, selective autophagy and diverse signaling pathways, is a new substrate of parkin^38^. Parkin interacts with the substrate pyruvate kinase M2 (PKM2) to regulates the glycolysis pathway and affects the cell metabolism^39^. CHOP was identified as a parkin substrate. Parkin blunts excessive CHOP to prevent ER stress-induced cell death and adverse cardiac ventricular remodeling^40^. Parkin inactivation leads to accumulation of the parkin interacting substrate (PARIS, ZNF746) that plays an important role in dopamine cell loss^41^. Substrates of parkin also include CDCrel-1, synphilin, and cyclin E. Parkin catalyzes the binding of ubiquitin to these substrates^42,43^. Ubiquitination, phosphorylation, and acetylation are reversible post-translational modifications that can trigger or inhibit the activity of parkin^14^. The phosphorylation of Ser131, Ser101, Ser127 and Ser378 led to the abnormal aggregation of parkin without specifically affecting its E3 ubiquitin ligase activity^44^. However, the phosphorylation of Tyr143 inhibited parkin E3 ubiquitin ligase activity during PD in vivo and in vitro^45,46^. Additionally, parkin regulates its own ubiquitination by the Lys48 proteasome-dependent ubiquitin chain^12,14^. The precise location and function of parkin during ubiquitination remain to be determined.

The excessive production of NO in PD causes nitrative stress damage to nigral dopaminergic neurons. Nitrification-induced protein thiol nitrosylation was shown to be involved in the pathogenesis of PD^16^. S-nitrosylation inhibited the E3 ubiquitin ligase activity of parkin, which led to the decrease of the ubiquitination and degradation of substrates, abnormal protein clearance, and, finally, the death of dopaminergic neurons^17,22^. SNO-parkin was detected in brains from both animal PD models and human PD patient^22^. Parkin dysfunction caused by nitrosative stress suggests a probable link between free radical production and abnormal protein accumulation in PD. In this study, GSNO or MPP^+^ treatment elevated the level of oxidized NO, resulting in the S-nitrosylation of parkin. Meanwhile, the expression of DMT1, iron uptake and cell death were significantly increased also.By cell viability detection, we can infer that DMT1 is degraded by parkin and could not transfer excessive iron ions into cells, which will change the cell viability. However, GSNO plays an impired role in several ways, it could not reflect protective effect of parkin in cells with GSNO treatment. The effect of parkin mutant, which is related to the function of S-nitrosylation, on cell viability could not be reflected, too.

The production of SNO-containing proteins led to changes in protein conformation, enzyme activity, protein–protein interactions and cell localization^47,48^, which then affected protein function^16^. Five thiolation sites were found at cysteine residues (amino acids 59, 95, 182, 212 and 377) in parkin protein. But it was unclear whether mutations at these sites had an effect on the regulation of ubiquitination^49^. Cys241, Cys260^22^ in the RING1 domainand and Cys323^21^ in the IBR domain are S-nitrosylated sites in parkin. Based on this, individual parkin mutants (Cys241A, Cys260A, and Cys323A) by site-directed mutagenesis were generated. However, whether there is any other S-nitrosylation site in parkin is still unclear.

In conclusion, the current study showed that the S-nitrosylation of parkin inhibited its E3 ubiquitin ligase activity, preventing the ubiquitination and degradation of DMT1. Increased DMT1 levels resulted in enhanced iron uptake, which aggravated iron accumulation in PD. Our study suggested that S-nitrosylation of parkin was essential for cell survival, which provided a new target for the prevention and treatment of PD.

## Materials and methods

### Cell culture

SH-SY5Y cells and the HEK293T cells were purchased from the Chinese Academy of Sciences Cell Bank. SH-SY5Y cells were cultured in RPMI 1640 medium (Gibco, Grand Island, NY, USA) containing 15% fetal bovine serum (FBS; Hyclone, Logan, UT, USA) and 1% penicillin–streptomycin (Gibco, Grand Island, NY, USA). HEK293T cells were cultured in DMEM high glucose medium (HyClone, MA, USA) containing 10% FBS (Hyclone, Logan, UT, USA) and 1% penicillin–streptomycin (Gibco, Grand Island, NY, USA). Both cell lines required a culture environment containing 5% CO_2_ at 37 °C.

### Cell transfection and treatment

SH-SY5Y cells were seeded at a density of 1 × 10^5^/cm^2^ in 6-well plates. When the cells grew to 70%–80% confluence, a pCMV6 vector that contained cDNA encoding parkin was transfected into cells1 (1 μg/well). The plasmid was mixed with the Lipofectamine 2000 (Thermo Fisher Scientific, Waltham, MA, USA) for 30 min. Then serum-free medium was added for 4 h, followed by serum-containing medium. The SH-SY5Y cell line transiently overexpressing parkin (Parkin-SY5Y cells) has been prepared. As a negative control, an empty vector was transfected into cells.

SH-SY5Y cells were exposed for 24 h with 200 μM of GSNO (Santa Cruz Biotechnology, CA, USA), or glutathione (GSH; Santa Cruz Biotechnology, CA, USA), as the negative control. Cells were also exposed to 200 μM of MPP^+^ (Sigma, Ronkonkoma, NY, USA), or normal saline (NS, 0.9% saline), as the blank control. In some cases, 1 mM of the broad spectrum NOS inhibitor NG-nitro-L-Arginine (L-NNA; Enzo Biochem, NY, USA) was added. In other cases, 100 μM of Fe^2+^ was added.

### In vivo experiment

The experimental animals are provided by Qingdao Institute for drug control. The operations on animals are in accordance with the rules of experimental animals. Male C57BL/6 mice aged 8–10 weeks were randomly divided into six groups: control group, 2 h group, 4 h group, 24 h group, 48 h group and72 h group, with an average of 6 mice in each group. 1-Methyl-4-phenyl-1,2,3,6-tetrahydropyridine (MPTP) was dissolved in normal saline to prepare a concentration of 6.25 mg / ml. Mice were intraperitoneally injected with MPTP (20 mg/kg) once every two hours, while the control group was intraperitoneally injected with the same volume of normal saline, a total of 4 times. After the last injection, at 2 h, 4 h, 24 h, 48 h, 72 h, the substantia nigra and striatum were collected for tissue protein extraction.

### Biotin-switch assay for detection of S-nitrosylated protein

S-nitrosylated protein was detected using a biotin switch assay according to the instructions of a S-nitrosylated Protein Detection Assay Kit (Cayman Chemical, Ann Arbor, MI, USA)^17^. Firstly, the free sulfhydryl group in the sample was blocked with methyl methanethiol sulfonate (MMTS). Secondly, the excess blocking agent was removed by acetone precipitation, and the thiol nitroso group (-SNO) was selectively reduced to free thiol by ascorbate. Finally, biotin-HPDP was added to label the newly synthesized thiol group. The biotin-labeled protein was adsorbed by streptavidin-agarose, subjected to denaturing polyacrylamide gel electrophoresis (SDS-PAGE).

### Western blotting analysis

The collected cell samples were lysed in RIPA lysis buffer (ComWin Biotech Co, Beijing, China) prepared with protease inhibitors (Roche, Basel, Switzerland), and then were placed on ice for 30 min. The lysed cells were centrifuged at 12,000×*g* for 20 min at 4 °C, and the supernatant were collected. Protein concentrations were determined by the BCA Protein Assay Kit (Thermo Fisher Scientific, CA, USA). The supernatant (20 μg/well) was mixed with 5 × SDS-PAGE loading buffer and boiled in a 100 °C water bath for 5–10 min. The protein samples were subjected to gel electrophoresis and then transferred to PVDF membranes. After blocking with 10% skimmed milk powder for 2 h at room temperature, the membranes were incubated with anti-parkin antibody (1:1000, Millipore, Massachusetts, USA), anti-DMT1 antibody (1:800, OriGene, Rockville, MD, USA), anti-β-actin antibody (1:10,000, Bioss, Woburn, MA, USA), anti-HA antibody (1:2000, Beyotime Biotechnology, Shanghai, China), anti-Myc antibody (1:2000, Beyotime Biotechnology, Shanghai, China) and anti-Flag antibody (1:2000, Beyotime Biotechnology, Shanghai, China) overnight at 4 °C. Membranes were incubated with a corresponding secondary antibody (1:10,000, Bioss, Beijing, China) for 1 h at room temperature. Finally, blots were imaged using a BioSpectrum Imaging System (UVP, Upland, CA, USA) and quantified using ImageJ software.

### Pulse chase experiment

SH-SY5Y cells were seeded in a 12 well plate. When the cell density reached 60%, the cells were transfected with empty vector and parkin respectively. After 24 h, 10 μg/ml of Cycloheximide (CHX, Sigma, NY, USA), GSNO (200 μM) or GSH (200 μM) were added, and then the cells were collected at 0 h, 4 h, 8 h, 12 h, 24 h and 48 h. Western blot was used to detect the changes of DMT1 protein.

### NO level assay

NO levels were detected using the nitrite and nitrate concentrations according to the instructions of the Nitric Oxide Assay Kit (Abcam, Cambridge, MA, USA)^50^. The assay buffer was added to the collected cells and centrifuged to remove insoluble impurities. The enzyme cofactor and nitrate reductase were added to the supernatant of each well to convert the nitrate to nitrite. Then an enhancer and a DAN probe were added to the sample to react the nitrite with the fluorescent probe. The addition of NaOH enhanced the fluorescence yield, which was proportional to the total nitric oxide production. The fluorescence was measured at wavelengths of 360 nm and 450 nm using a microplate reader. Extrapolate sample readings from the standard curve plotted using the following equation:

$$ {\text{Sa}} = \left[ {{\text{corrected}}\;{\text{absorbance}} - \left( {y - {\text{intercept}}} \right)} \right]/{\text{slope}}{.} $$

Concentration of nitrate and nitrite in the test samples is calculated as:

$$ {\text{Nitrite}} + {\text{nitrate}}\;{\text{concentration}} = {\text{Sa}}/{\text{Sv}} \times {\text{D}}. $$

Sa = Amount of nitrate + nitrite in the sample well (conc). Sv = Sample volume added into the reaction well (µL). D = Sample dilution factor.

### Calcein loading of cells and ferrous iron influx assay

Calcein is a stable fluorescent molecule, which could be quenched rapidly once combining with ferrous iron^11,51,52^. After different treatment, cells were seeded on coverslips in the 24-well plate and washed with HBS three times. Next, cells were incubated with 1 µM calcein-AM for 30 min (Yeasen, Shanghai, China) at 37 °C. Then the slides were removed and fixed in a heated chamber connected to a perfusion apparatus. Calcein fluorescence was recorded at 488 nm excitation and 525 nm emission wavelengths. And 10 scans were performed every 3 min for about 7 s, and 1 mm ferrous iron was infused at the same time. Additionally, ascorbate acted as a chelator to maintain the iron in solution. The X-Y-T scan mode was used to obtain the data.

### Cell viability assay

Cell viability was detected using CellTiter-Glo Luminescent Cell Viability Assay Kit (G7570, Promega, Madison, WI, USA) according to the manufacturer’s protocol. Cells were seeded in a 96-well plate. 24 h later, cells are treated in various ways according to “Cell transfection and treatment” as mentioned before. The reagent was added directly to cells in culture medium with a volume equivalent to the amount of medium. Mix for 2 min, and after 10-min incubation, detect the emitted luminescence using a microplate reader (Molecular Device, M5, Sunnyvale, CA, USA).

### Ubiquitination assays

In ubiquitination assays, HEK293T cells were used. Cells were transfected with Flag-WT-parkin, Myc-DMT1, and HA-ubiquitin. Plasmid of parkin with three mutations, Flag-C241A-parkin, Flag-C260A-parkin and Flag-C323A-parkin, were transfected into HEK293T cells respectively or together. 24 h later, cells were treated with GSNO for additional 24 h. The details are the same as described in “Cell transfection”. 10 μM of proteasome inhibitor MG132 (ApexBIO, Houston, USA,) was added 8 h prior to cell collection to block the rapid degradation of polyubiquitinated proteins. The cell lysates were immunoprecipitated using anti-Myc antibody. The ubiquitination level of DMT1 was detected by anti-HA antibody.

### Statistical analysis

Prism 8.0 software (GraphPad Software Inc., La Jolla, CA, USA) was applied to analyze the data. When comparing two sets of data, *P* value is greater than 0.05, which obeying normal distribution. Independent Samples Test is selected. When multiple groups of data are compared, with the test of homogeneity of variance, *P* value is greater than 0.05. One-way ANOVA is selected. Besides, the ferrous iron influx and pulse chase assay were analyzed by Two-way Repeated Measures ANOVA. Data are normally distributed by Shapiro Wilk test (*P* value is greater than 0.05). According to the hypothesis of Mauchly's sphere, the variance is homogeneous (*P* value is greater than 0.05). The effect of interaction was not statistically significant. Data are shown as the mean ± SEM. *P* < 0.05 was considered statistically significant.

## Supplementary information

Supplementary Information.

## Data Availability

The data used to support the findings of this study are available from the corresponding author upon request.

## Abbreviations

PD: Parkinson's disease
DMT1: Divalent metal transporter 1
GSNO: S-nitrosoglutathione
MPP^+^: 1-Methyl-4-phenylpyridinium
MPTP: 1-Methyl-4-phenyl-1,2,3,6-tetrahydropyridine
NO: Nitric oxide
WT: Wild-type
IRP: Iron responsive protein
IRE: Iron regulatory element
WWP2: WW domain-containing protein 2
PKM2: Pyruvate kinase M2
FBS: Fetal bovine serum
GSH: Glutathione
L-NNA: NG-nitro-l-arginine
MMTS: Methyl methanethiol sulfonate
BSA: Bovine serum albumin
SNO-parkin: S-Nitrosylated parkin
