# Supplementary material for: The S-nitrosylation of parkin attenuated the ubiquitination of divalent metal transporter 1 in MPP+-treated SH-SY5Y cells
Source: Sci Rep. 2020 Sep 23;10:15542. doi: 10.1038/s41598-020-72630-2 (PMC7511936; doi:10.1038/s41598-020-72630-2)
Supplement: Supplementary file 1 — Supplementary Information. [file 41598_2020_72630_MOESM1_ESM.docx]

**The S-nitrosylation of parkin attenuated the ubiquitination of divalent metal transporter 1** **in MPP^+^-treated SH-SY5Y cells**

**Running title: The S-nitrosylation of parkin attenuated the ubiquitination of DMT1 in PD**

Yanmin Zhong^1#^, Xin Li^1, 2#^, Xixun Du^1^, Mingxia Bi^1^, Fengtong Ma^3^, Junxia Xie^1^, Hong Jiang^1*^

^1^State Key Disciplines: Physiology, Department of Physiology, Shandong Key Laboratory of Pathogenesis and Prevention of Neurological Disorders, School of Basic Medicine, Qingdao University, Qingdao 266071, China

^2^Office of Drug Clinical Trial Management, The Affiliated Hospital of Qingdao University, Qingdao 266003, China

^3^Grade 2017, Clinic Medicine, Medical College, Qingdao University, Qingdao 266071, China

^#^These authors contributed equally to this work.

^*^Correspondence: [hongjiang@qdu.edu.cn](mailto:hongjiang@qdu.edu.cn)


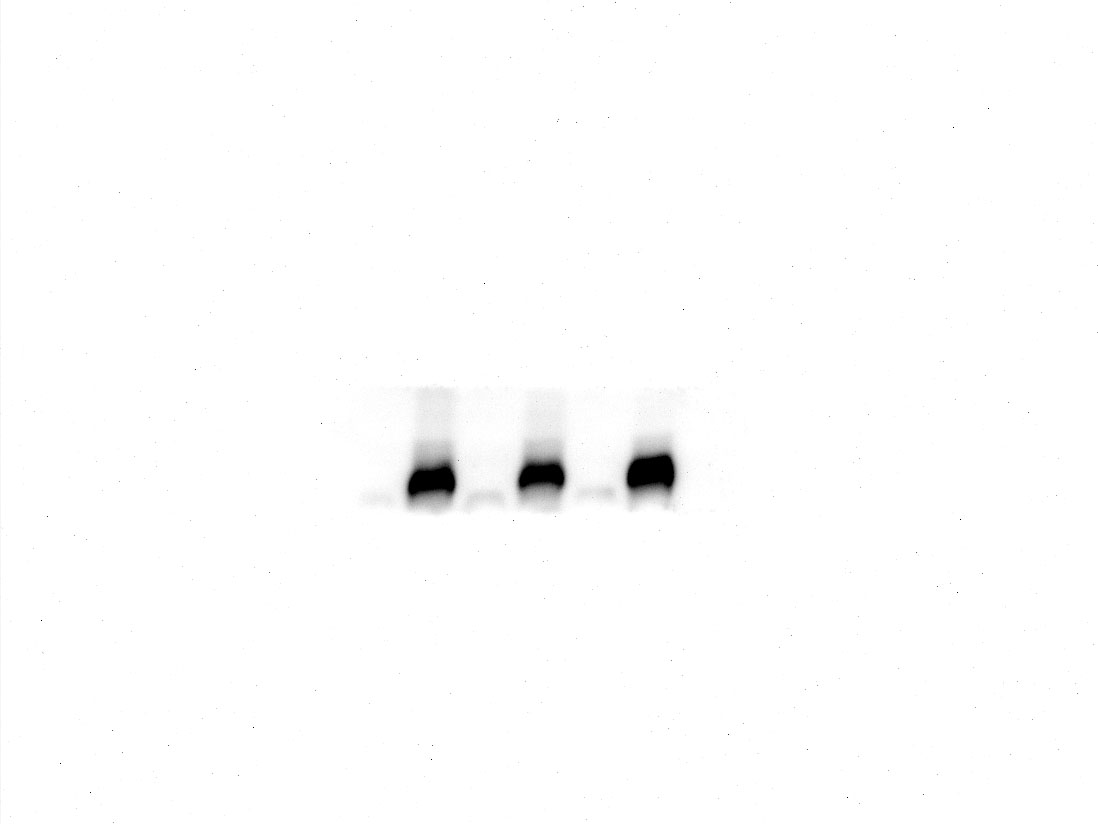


fig1a parkin


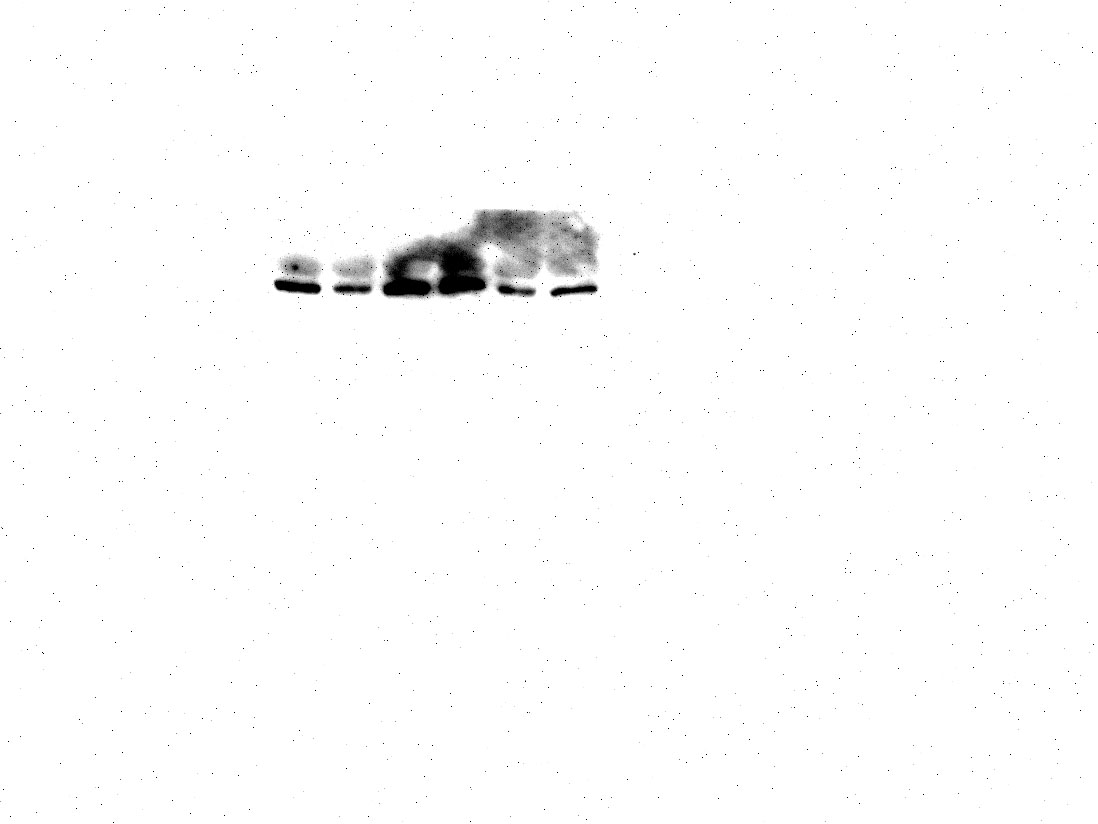


fig1a DMT1


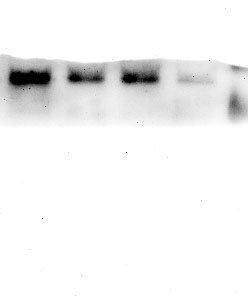


fig1a DMT1 (The image was obtained from repeated experiments, and was not shown in the manuscript.)


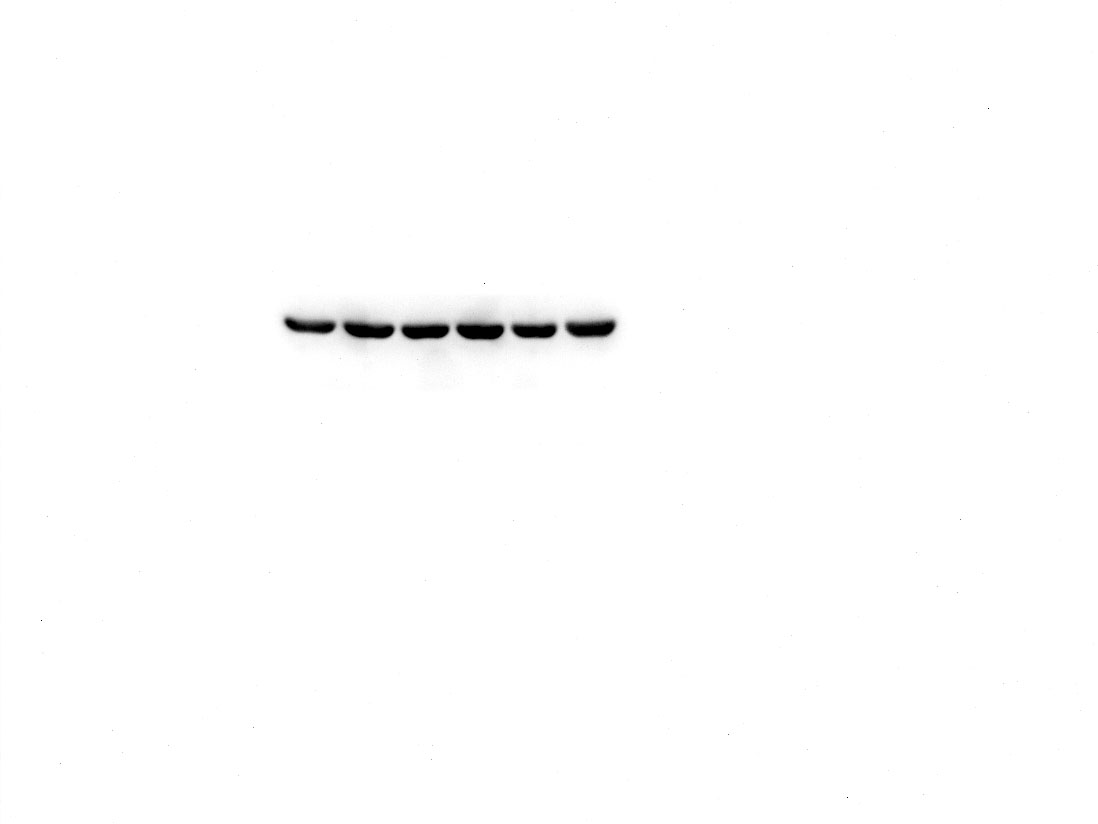


fig1a β-actin


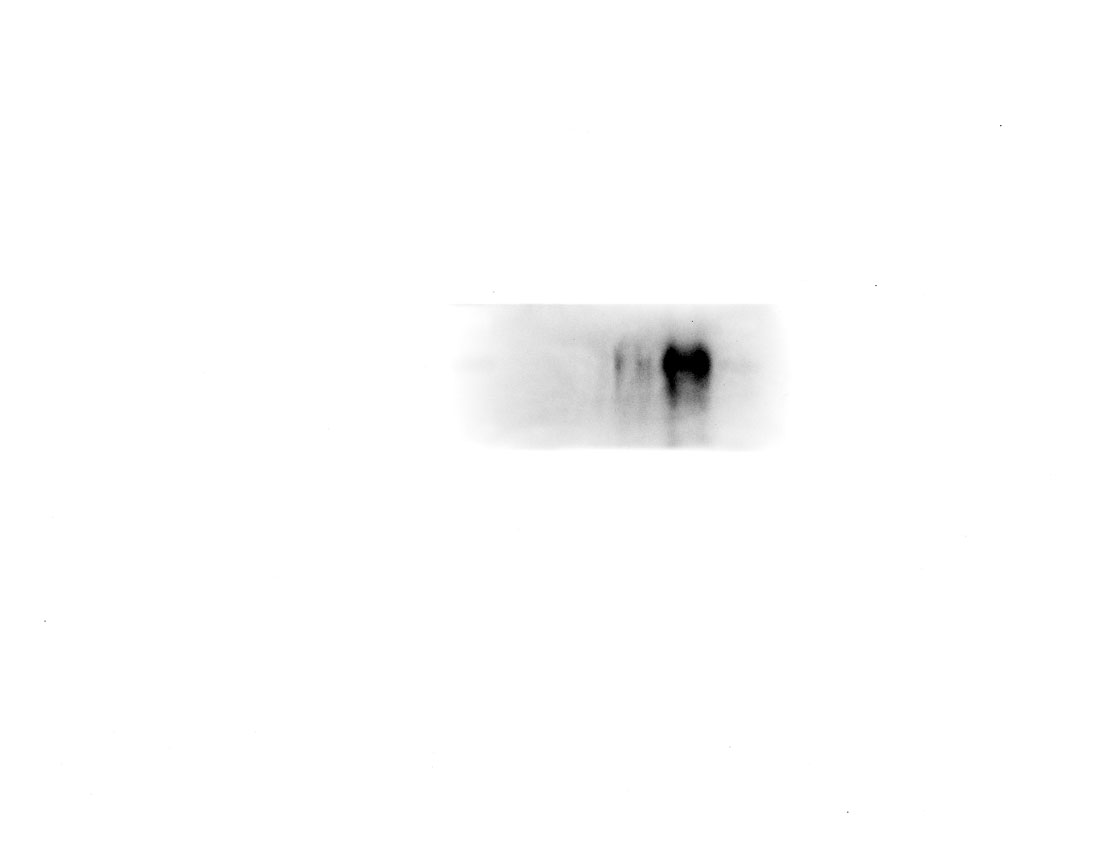


fig2a SNO-parkin


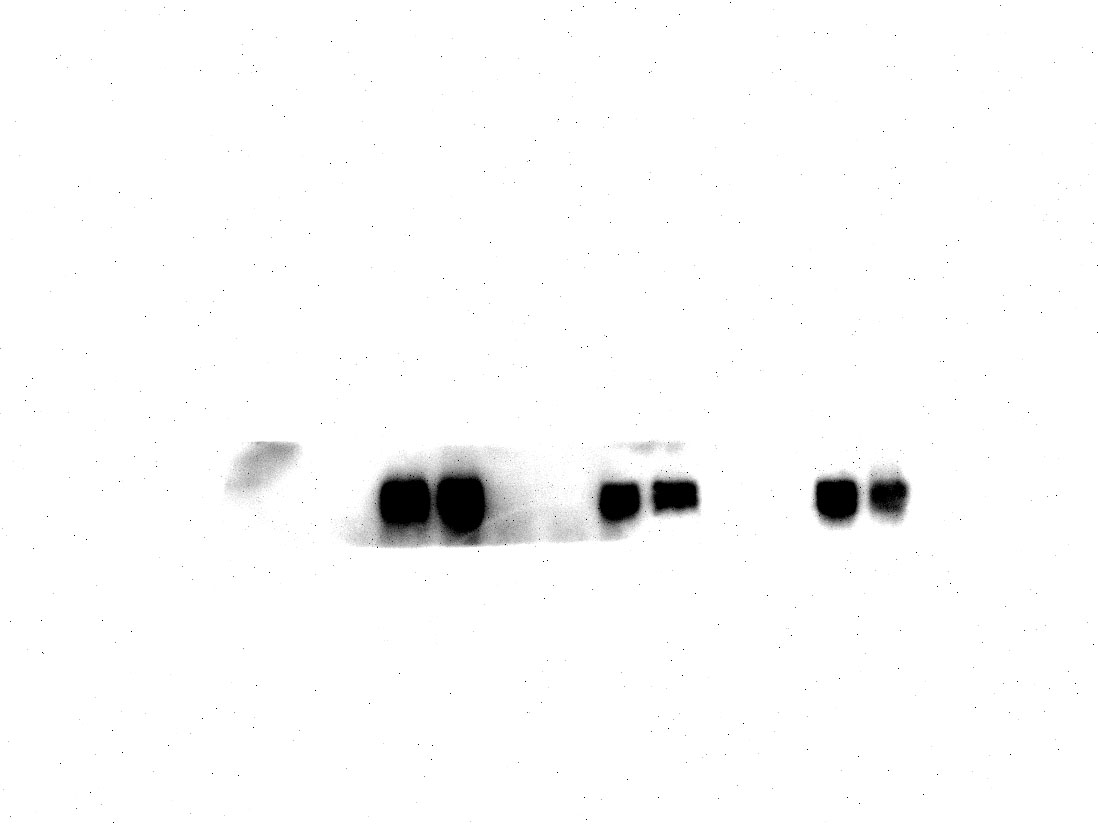


fig2a parkin (The image was obtained from repeated experiments and was not shown in the manuscript.)


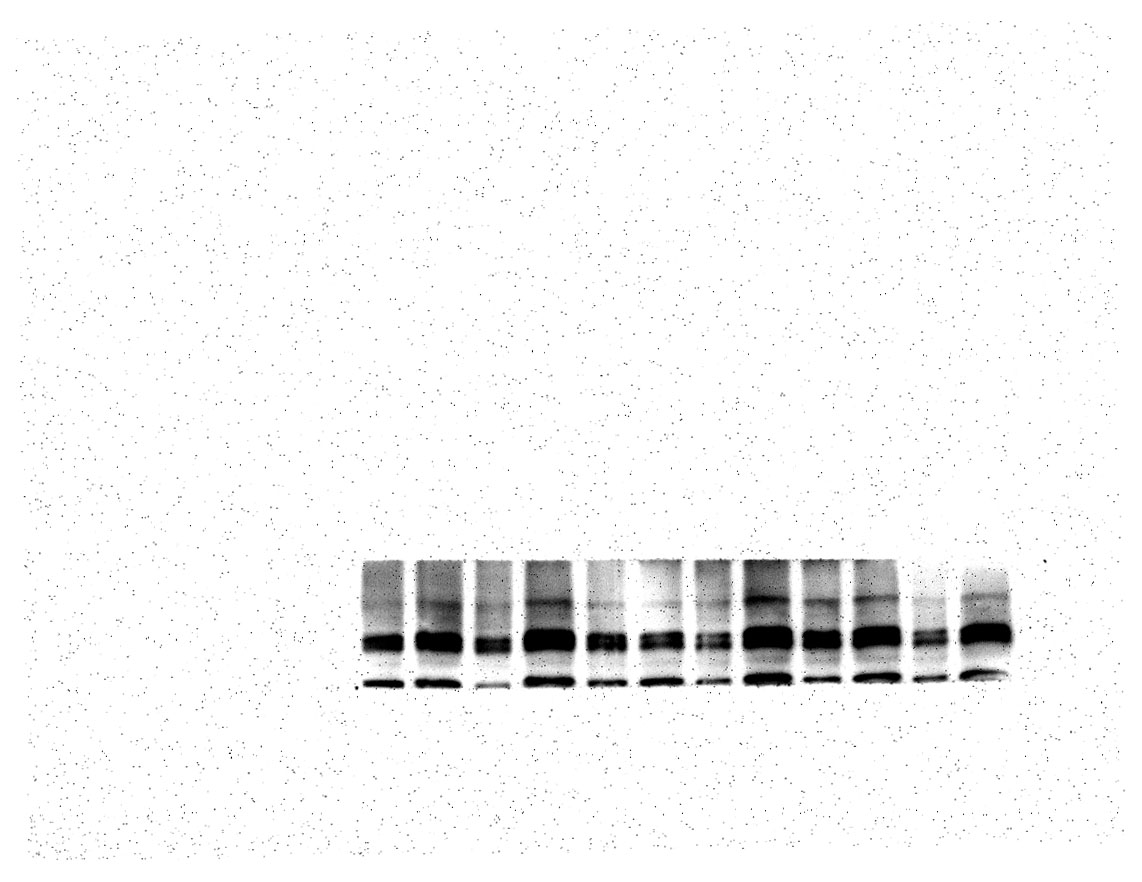


fig2a DMT1


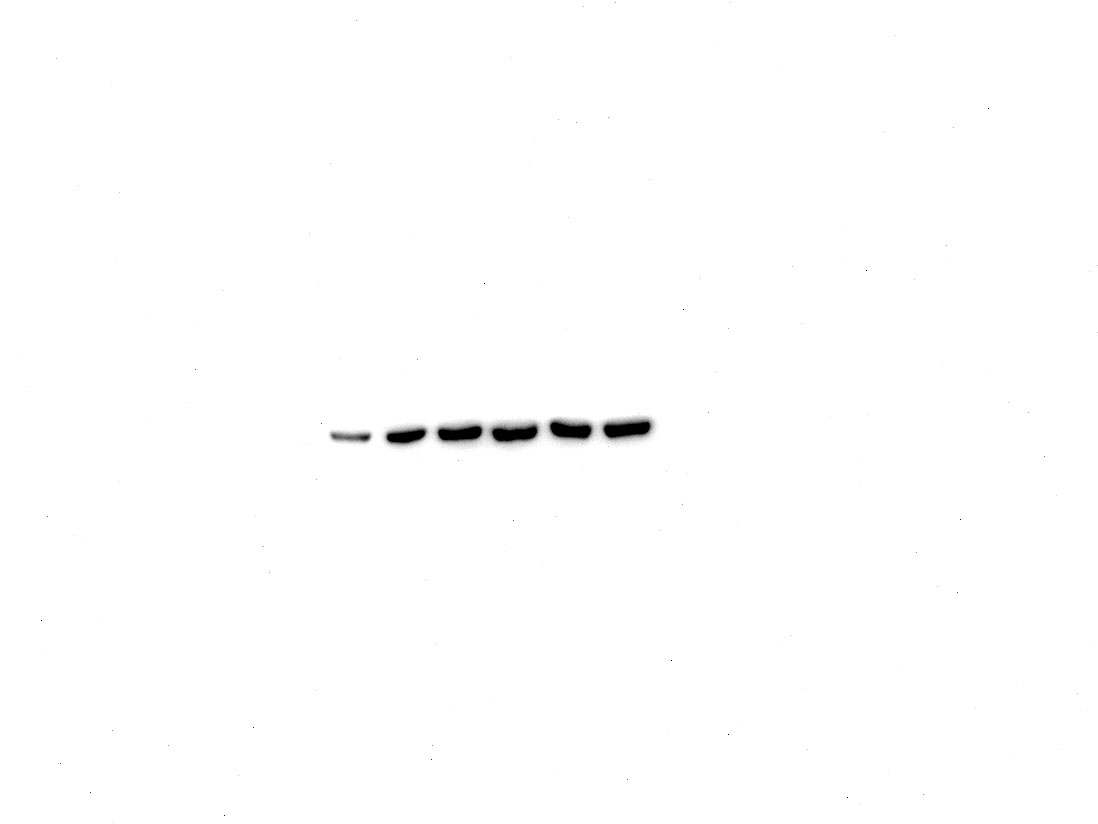


fig2a β-actin


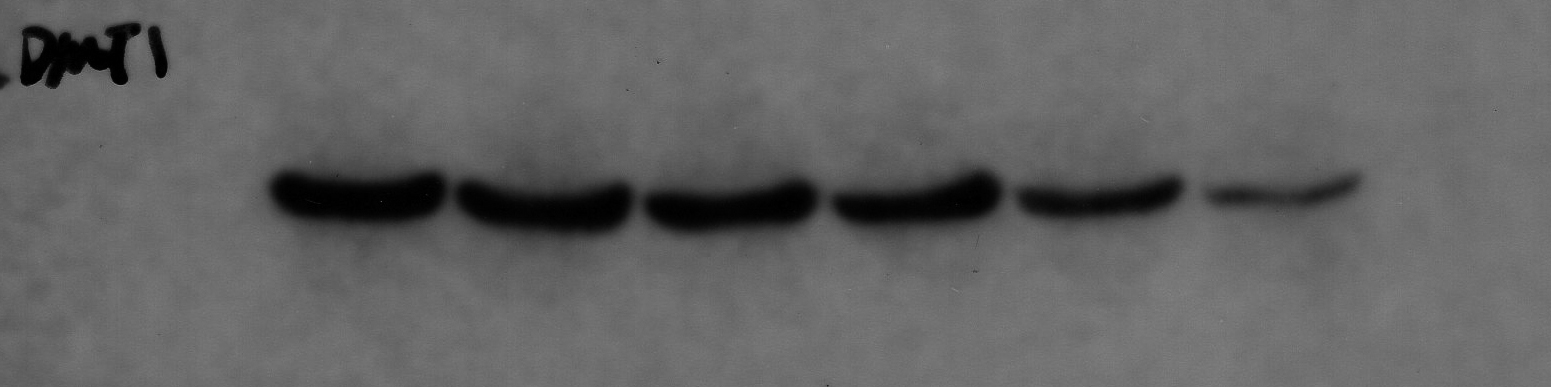


fig2d DMT1_1


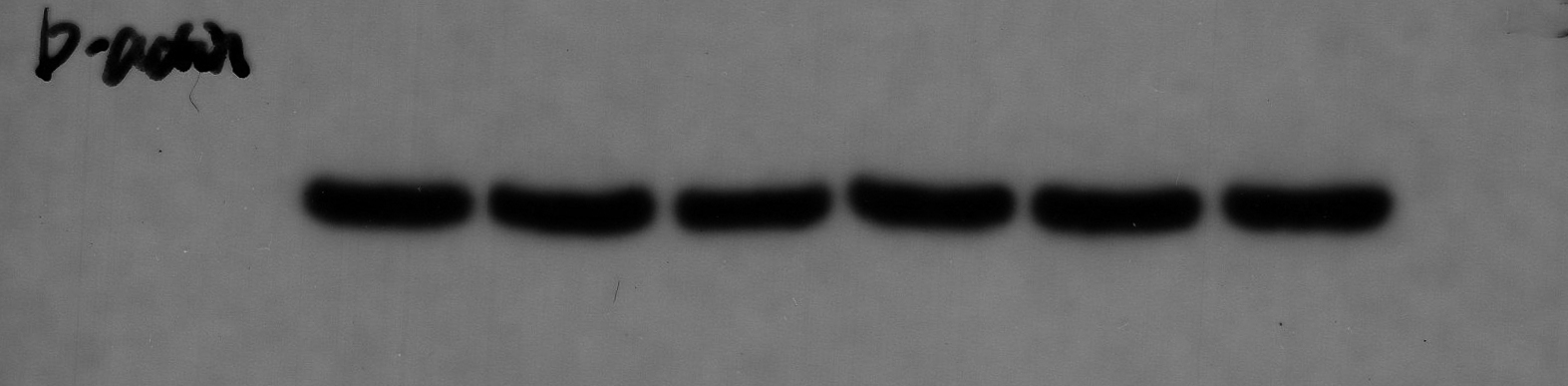


fig2d β-actin_1


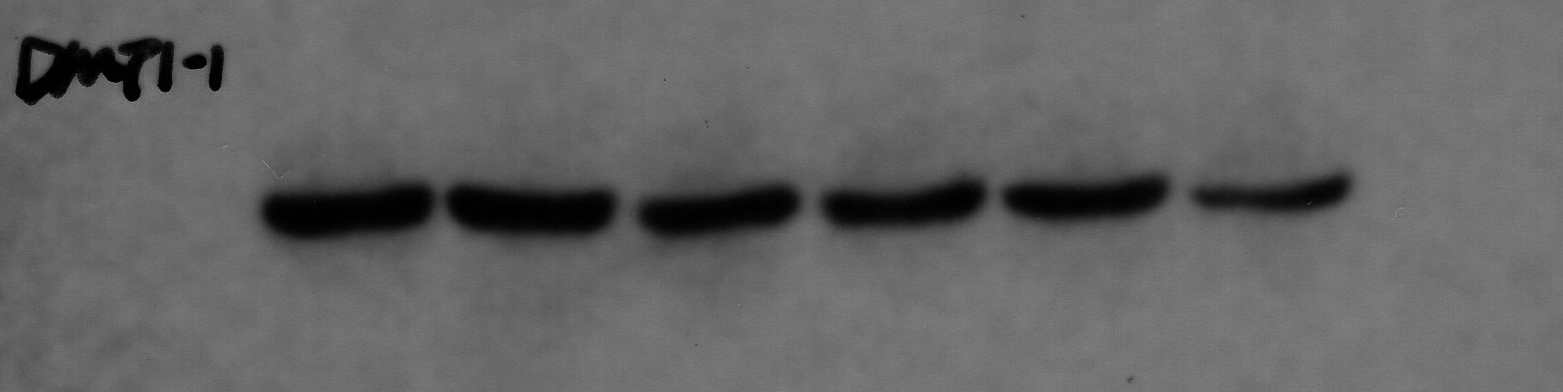


fig2d DMT1_2


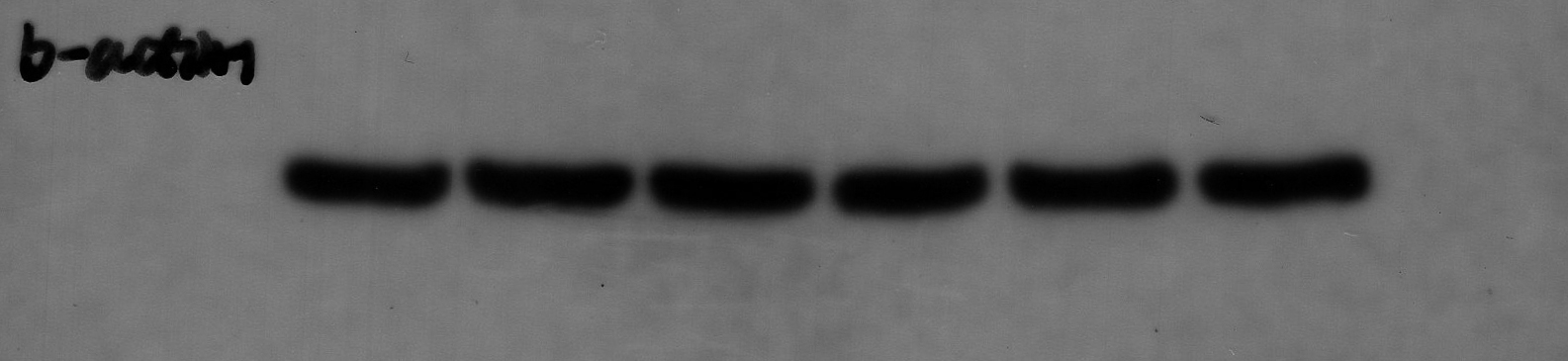


fig2d β-actin_2


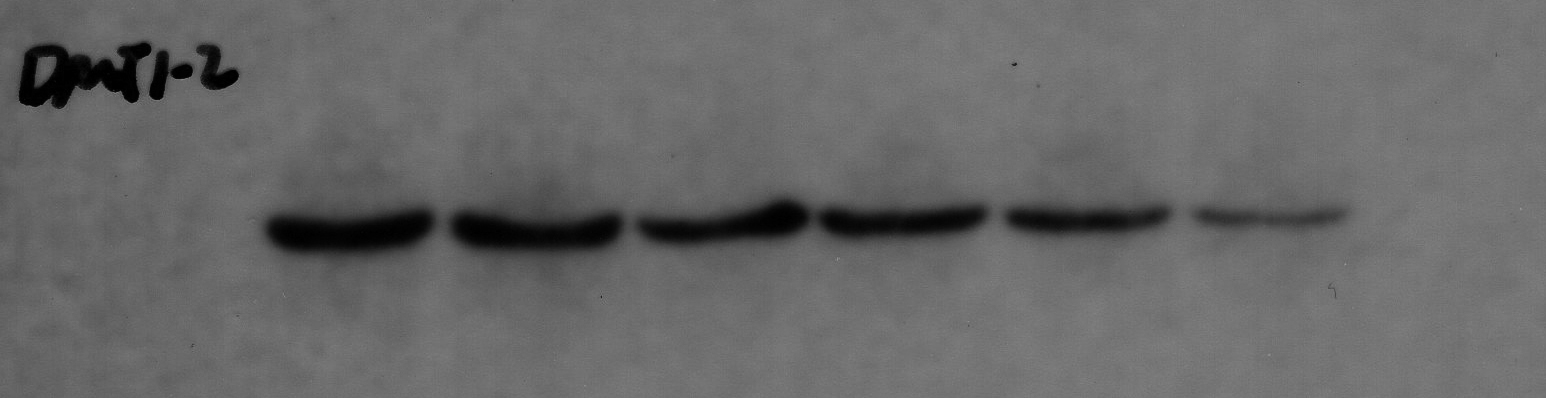


fig2d DMT1_3


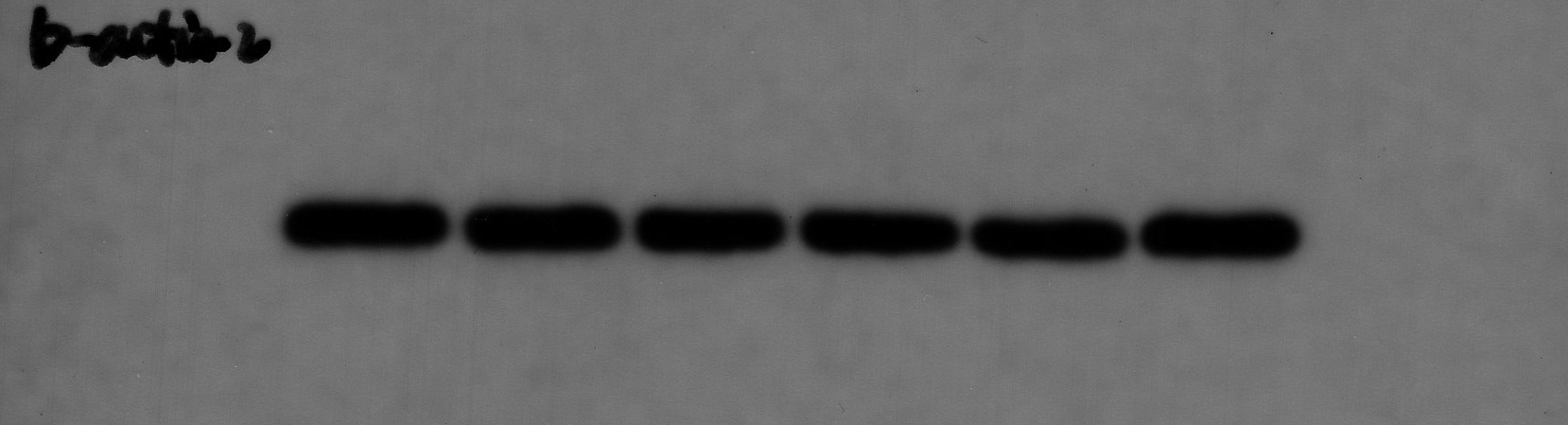


fig2d β-actin_3


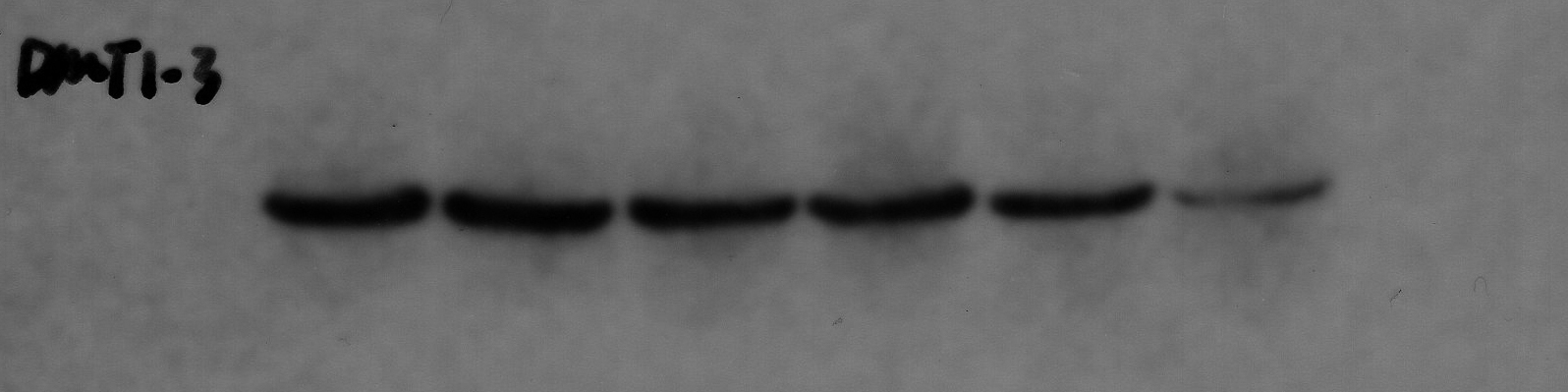


fig2d DMT1_4


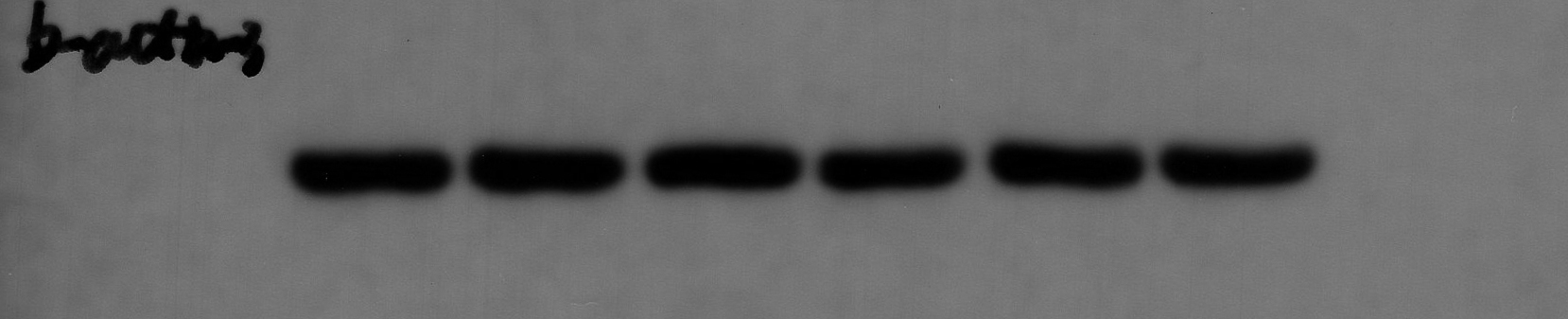


fig2d β- actin_4


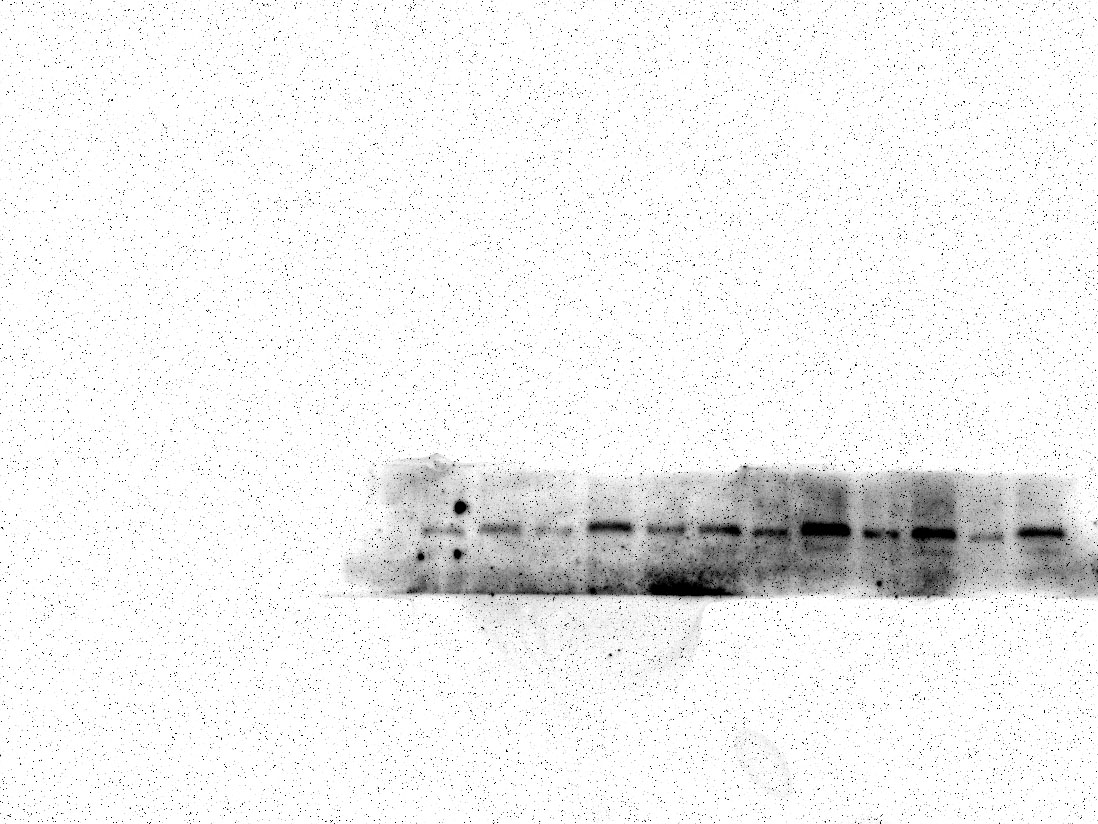


exposure time: 30 seconds


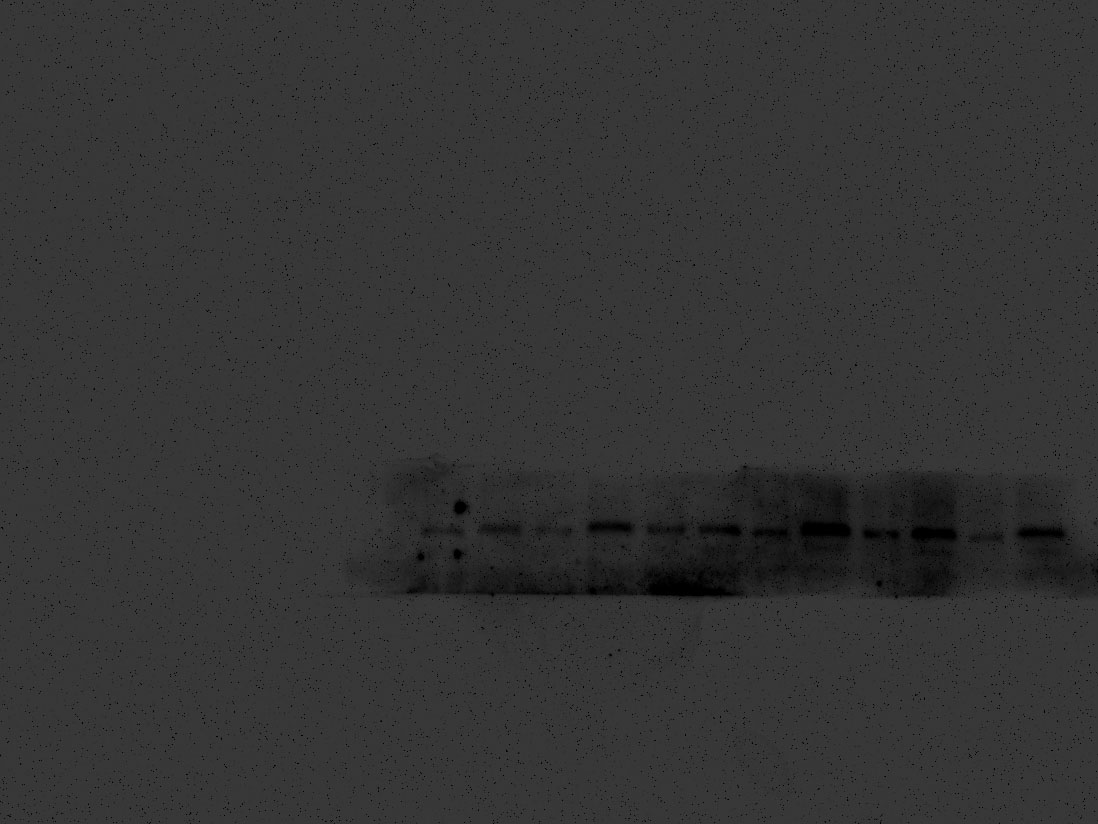


exposure time: 50 seconds

fig4a SNO-parkin (The images were obtained from repeated experiments with the different exposure time, which was not shown in the manuscript.)


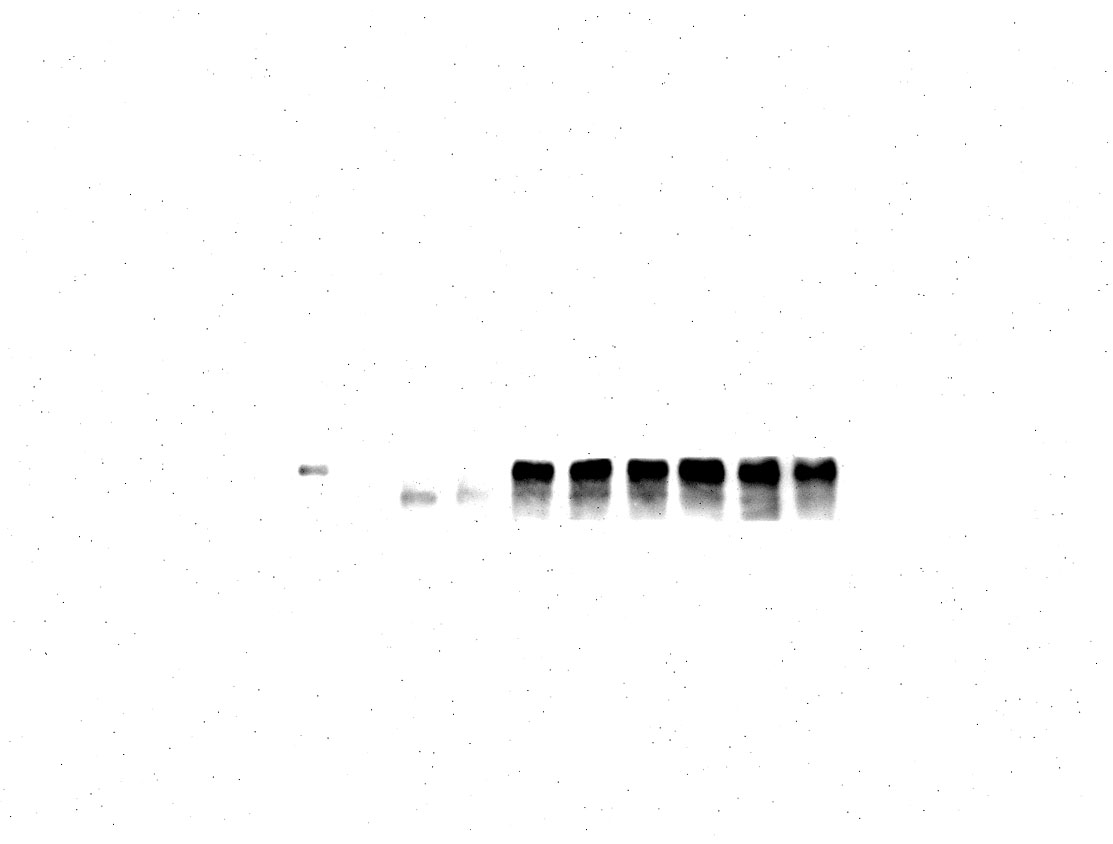


exposure time: 30 seconds


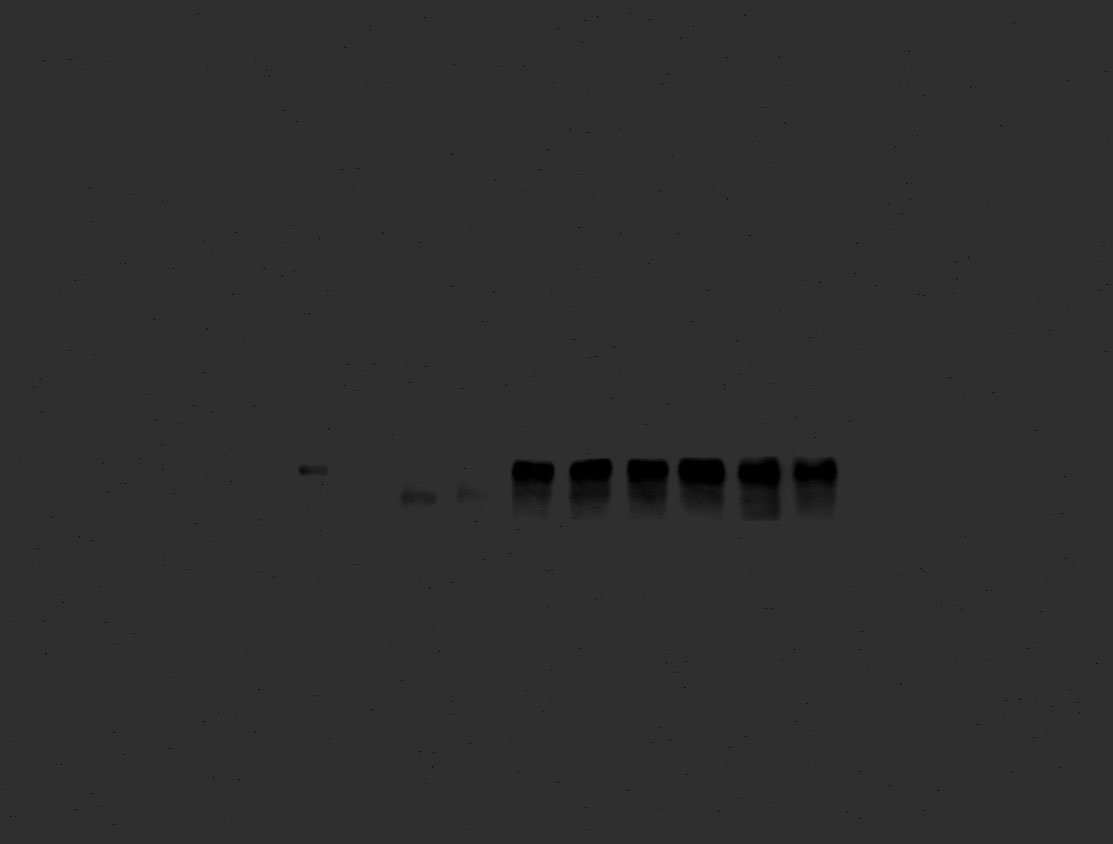


exposure time: 50 seconds

fig4a parkin (The two images are from the same experiment, but the exposure time is different.)


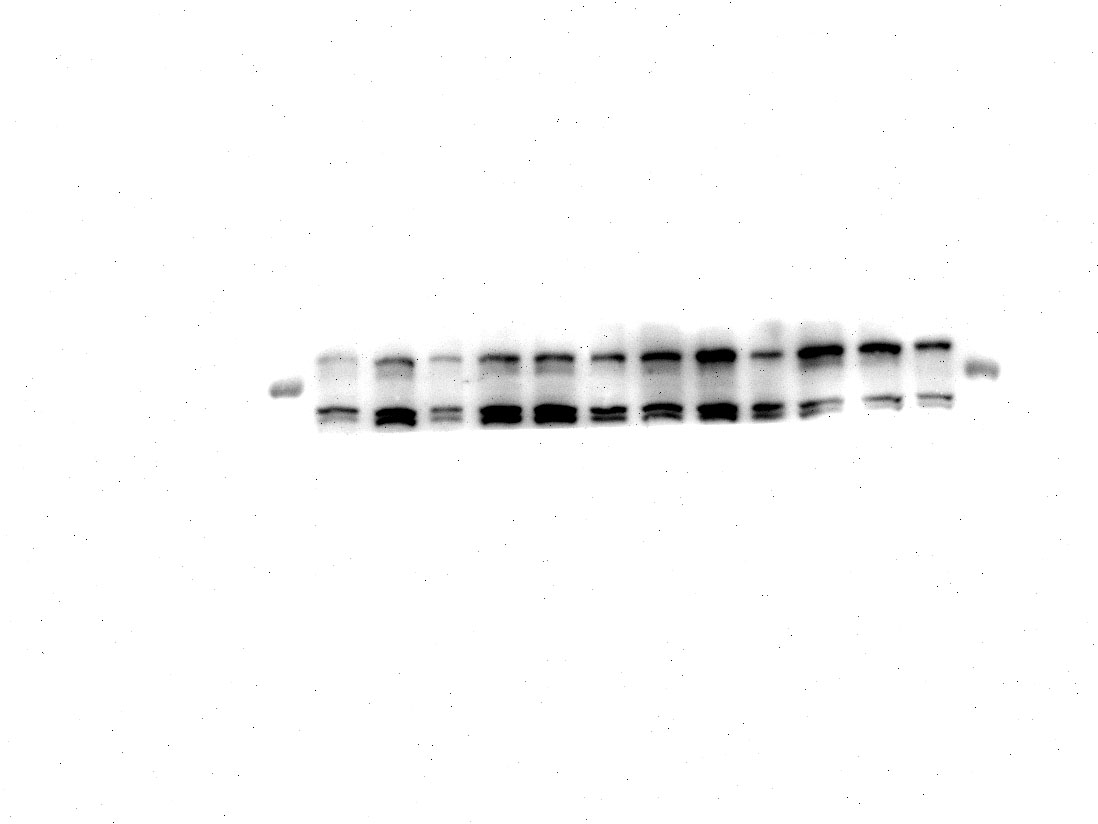


fig4a DMT1


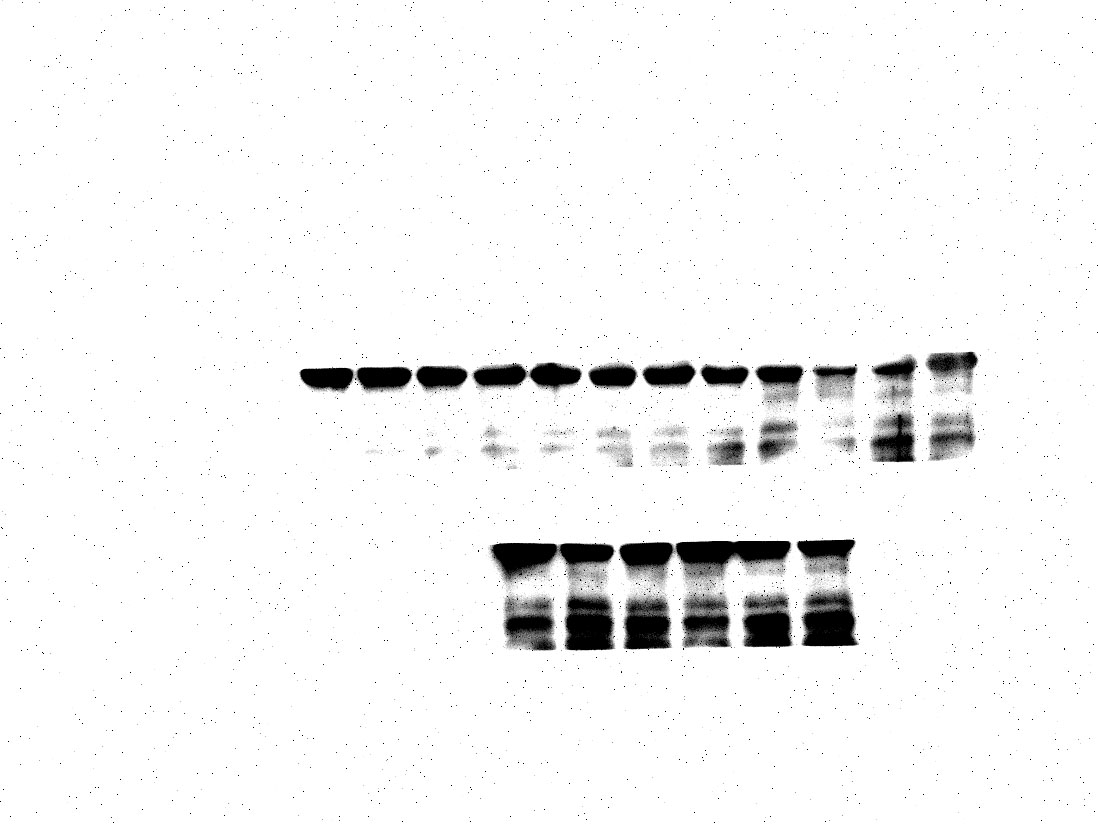


exposure time: 5 seconds


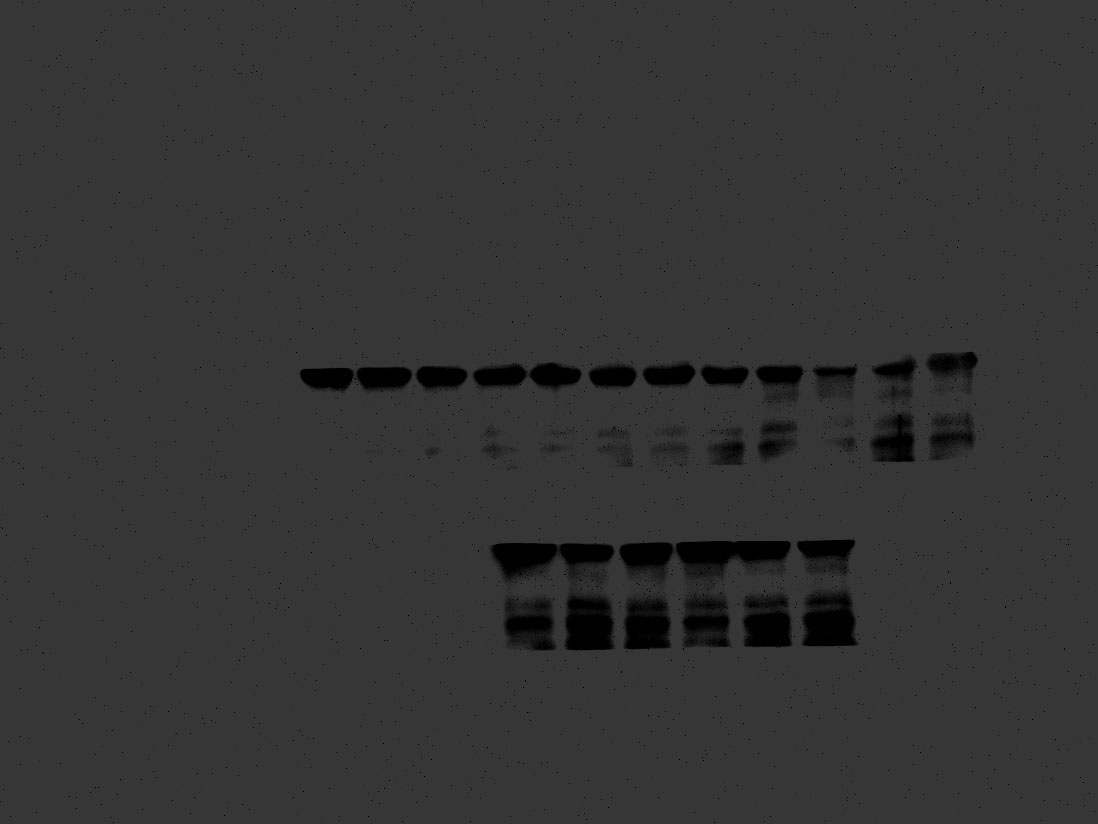


exposure time: 10 seconds

fig4a β-actin (The two images are from the same experiment, but the exposure time is different.)


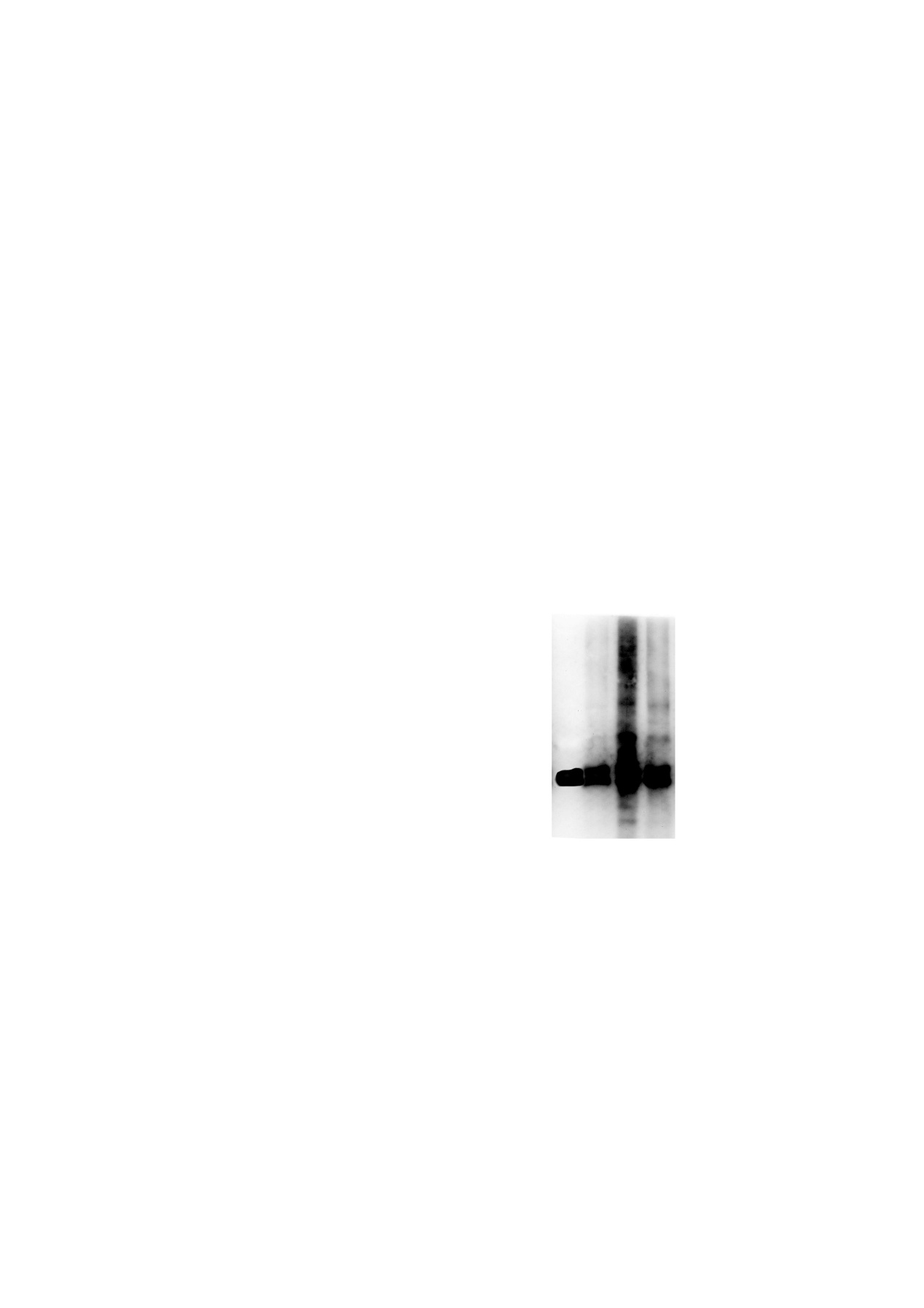


fig6a IP-Myc IB-HA


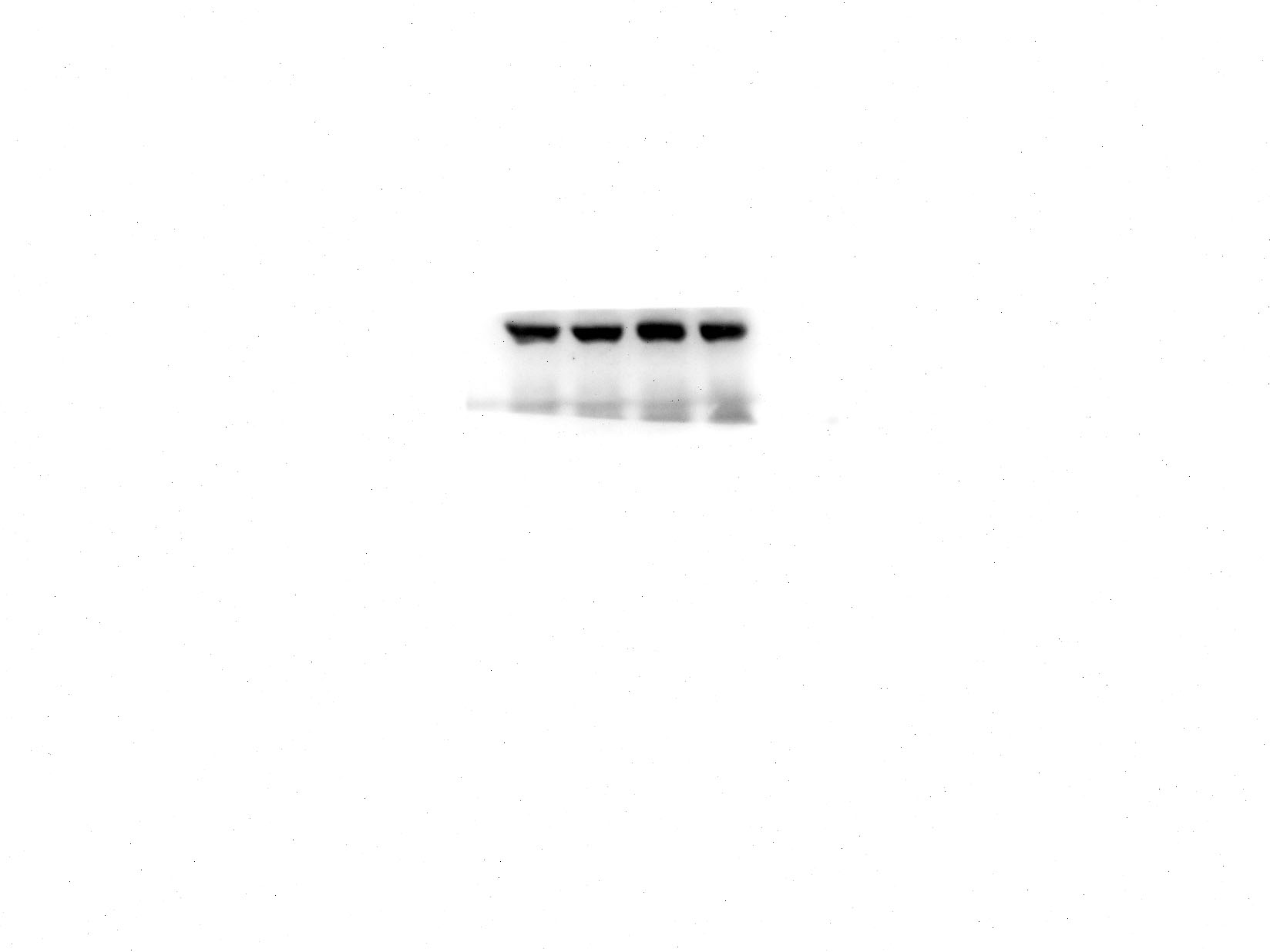


fig6a IP-Myc IB-Myc


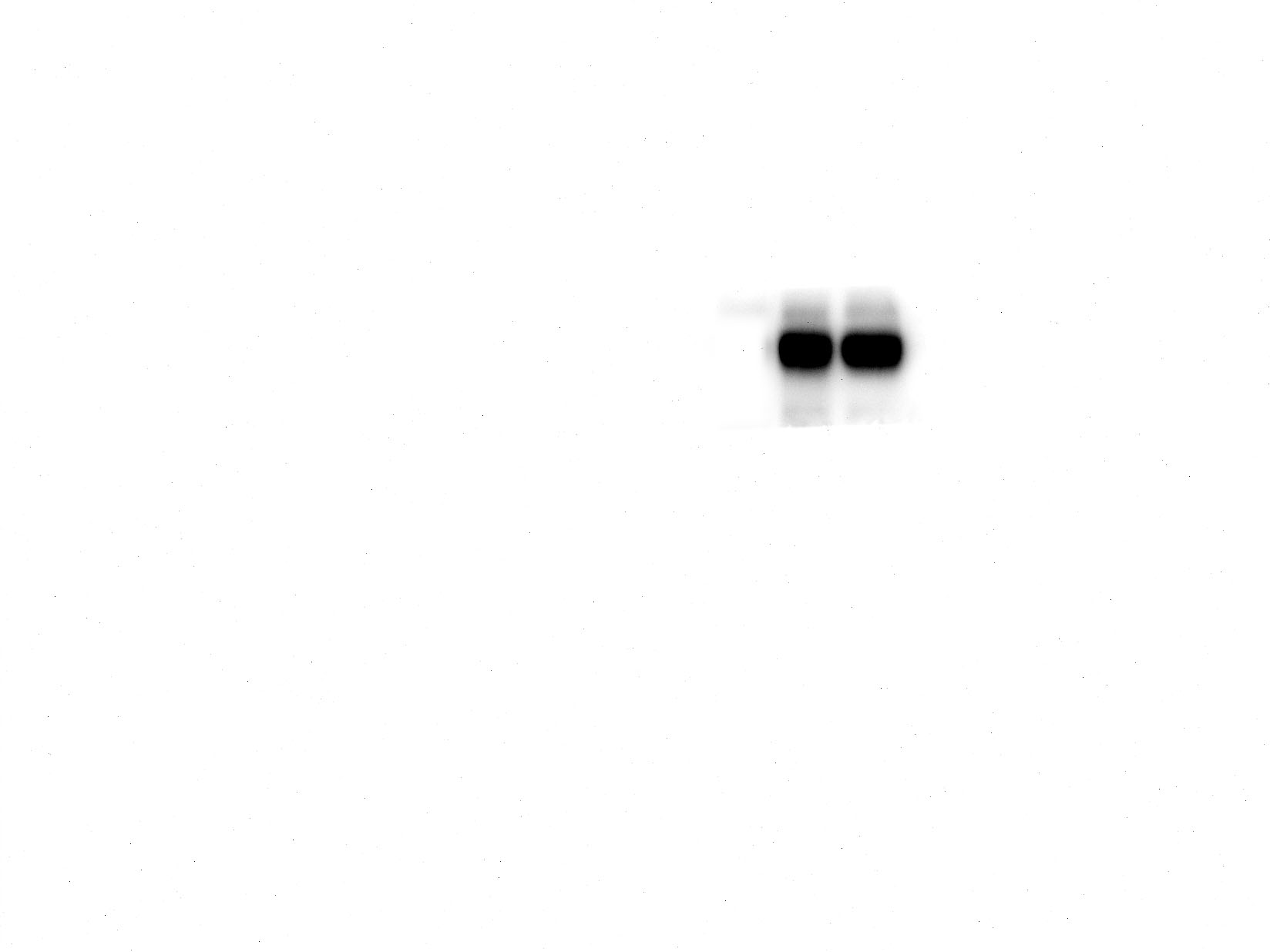


fig6a Input IB-flag


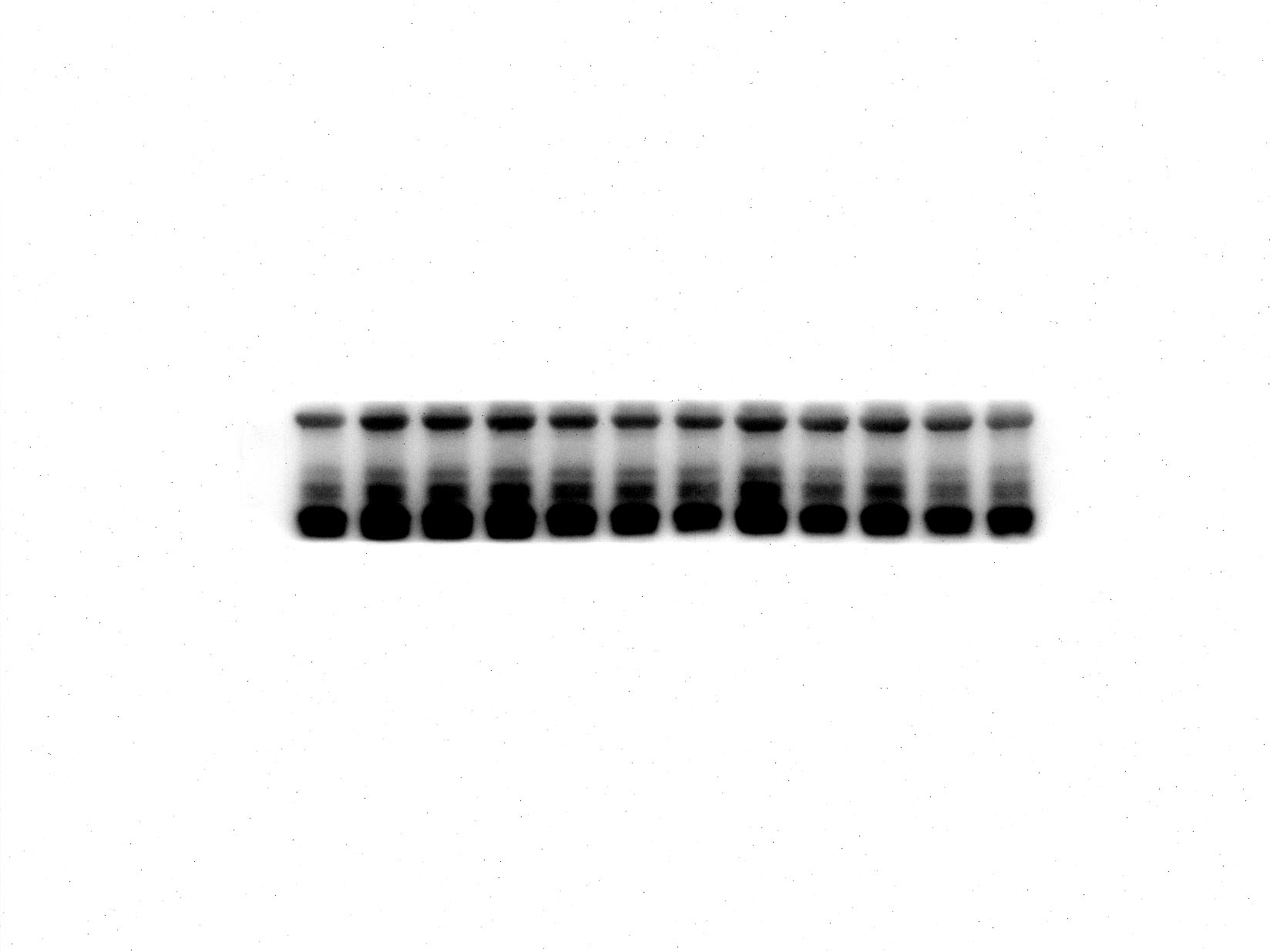


fig6a Input IB-myc


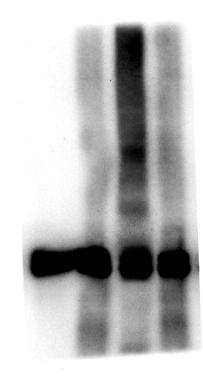


fig6b1 IP-Myc IB-HA


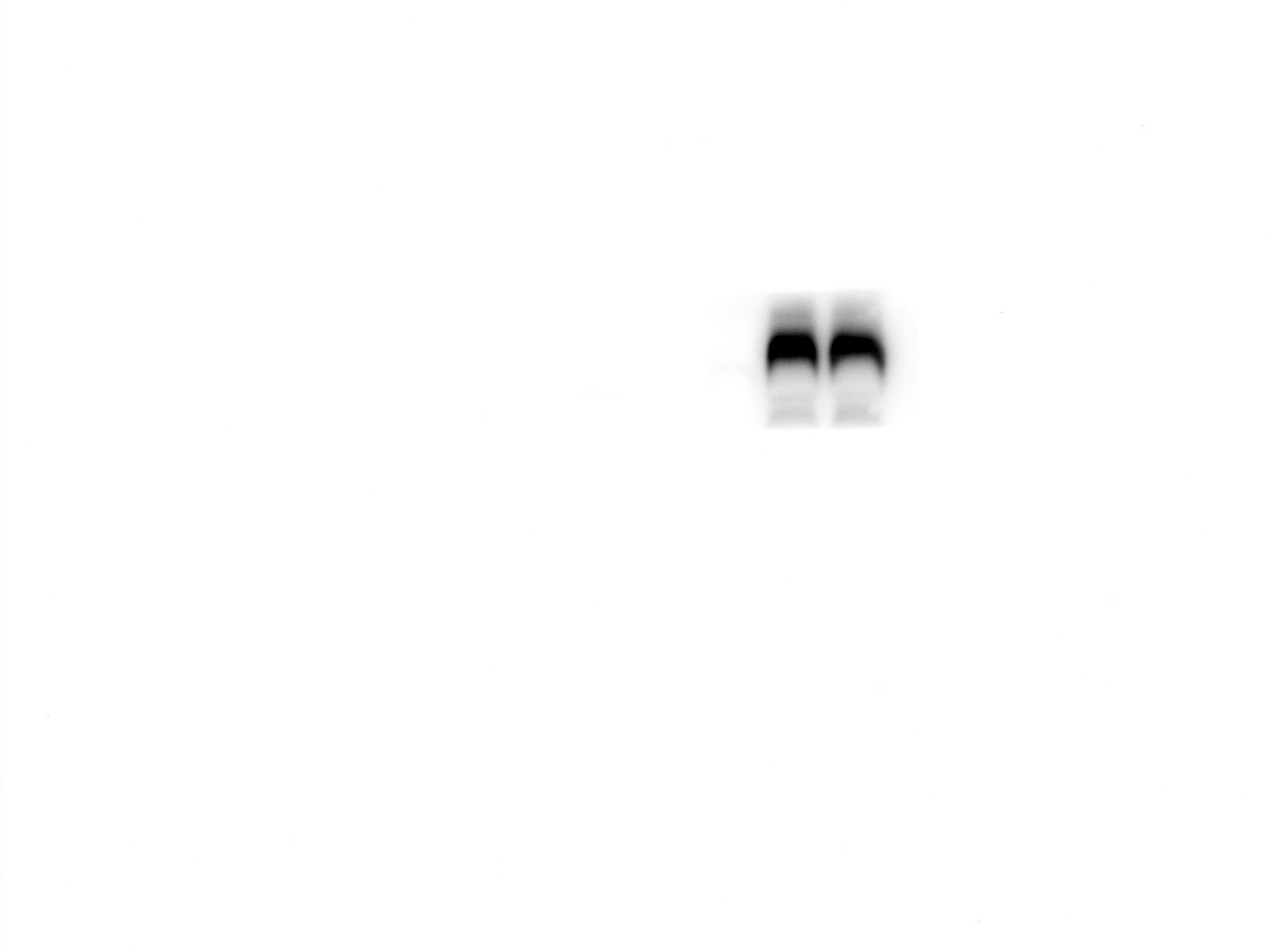


fig6b1 Input IB-flag


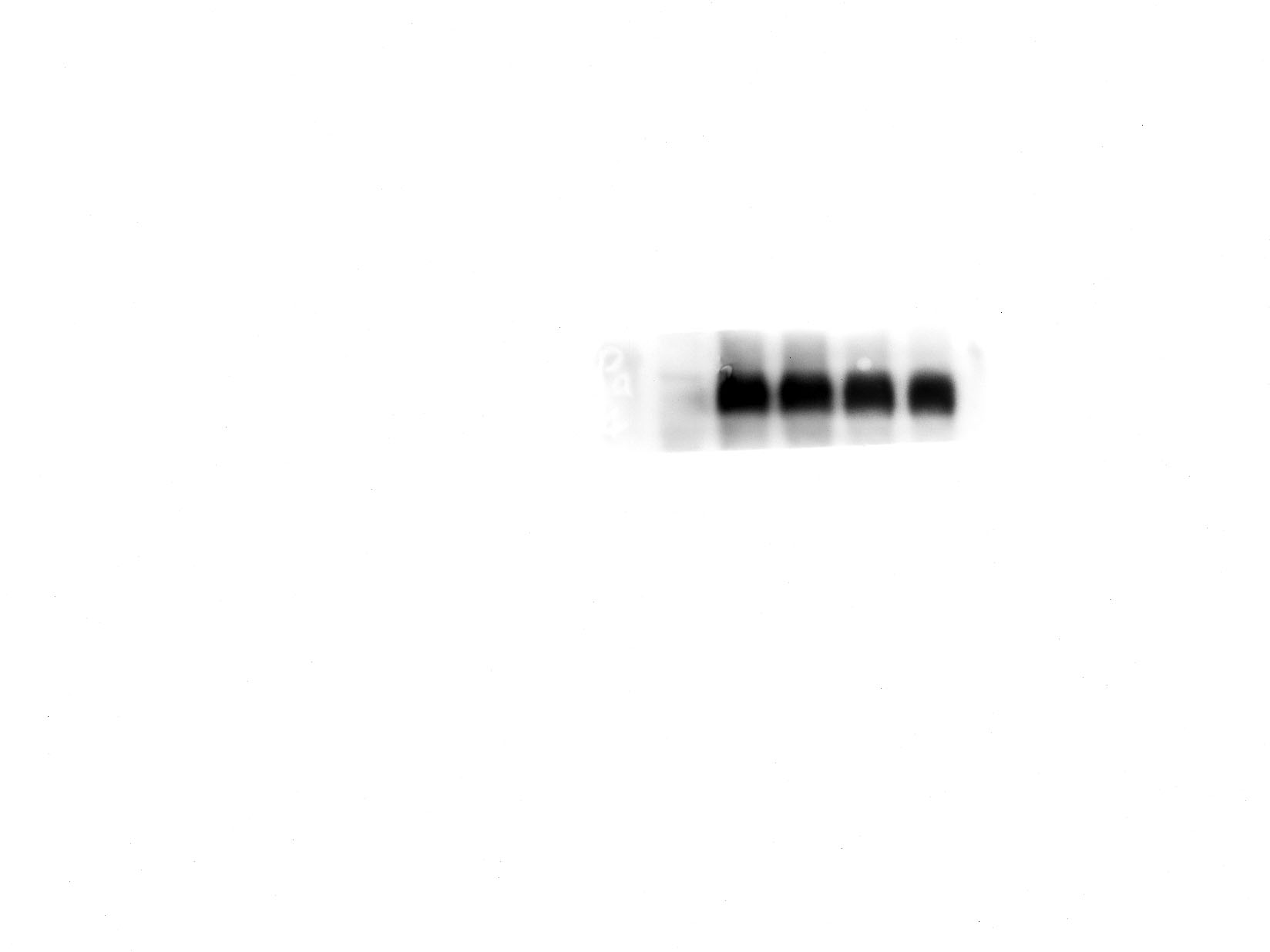


fig6b1 Input IB-Myc


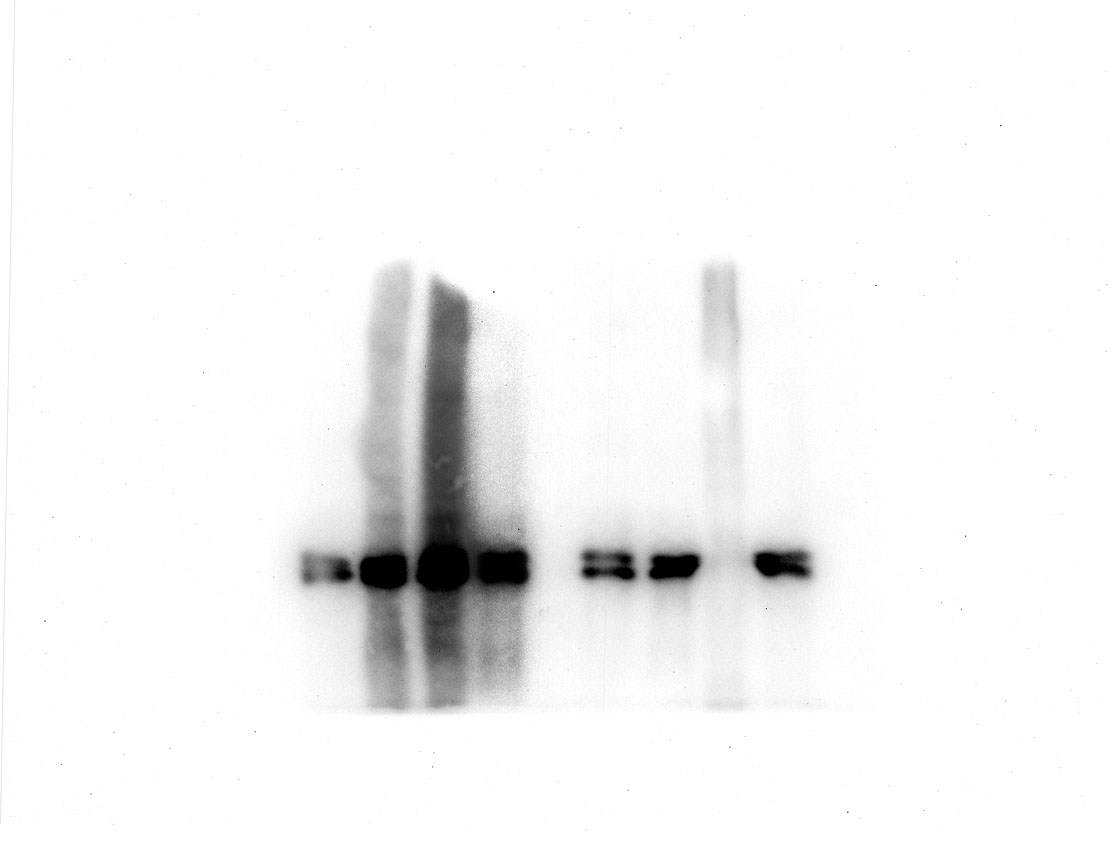


fig6b2 IP-Myc IB-HA (The image was obtained from repeated experiments and was not shown in the manuscript.)


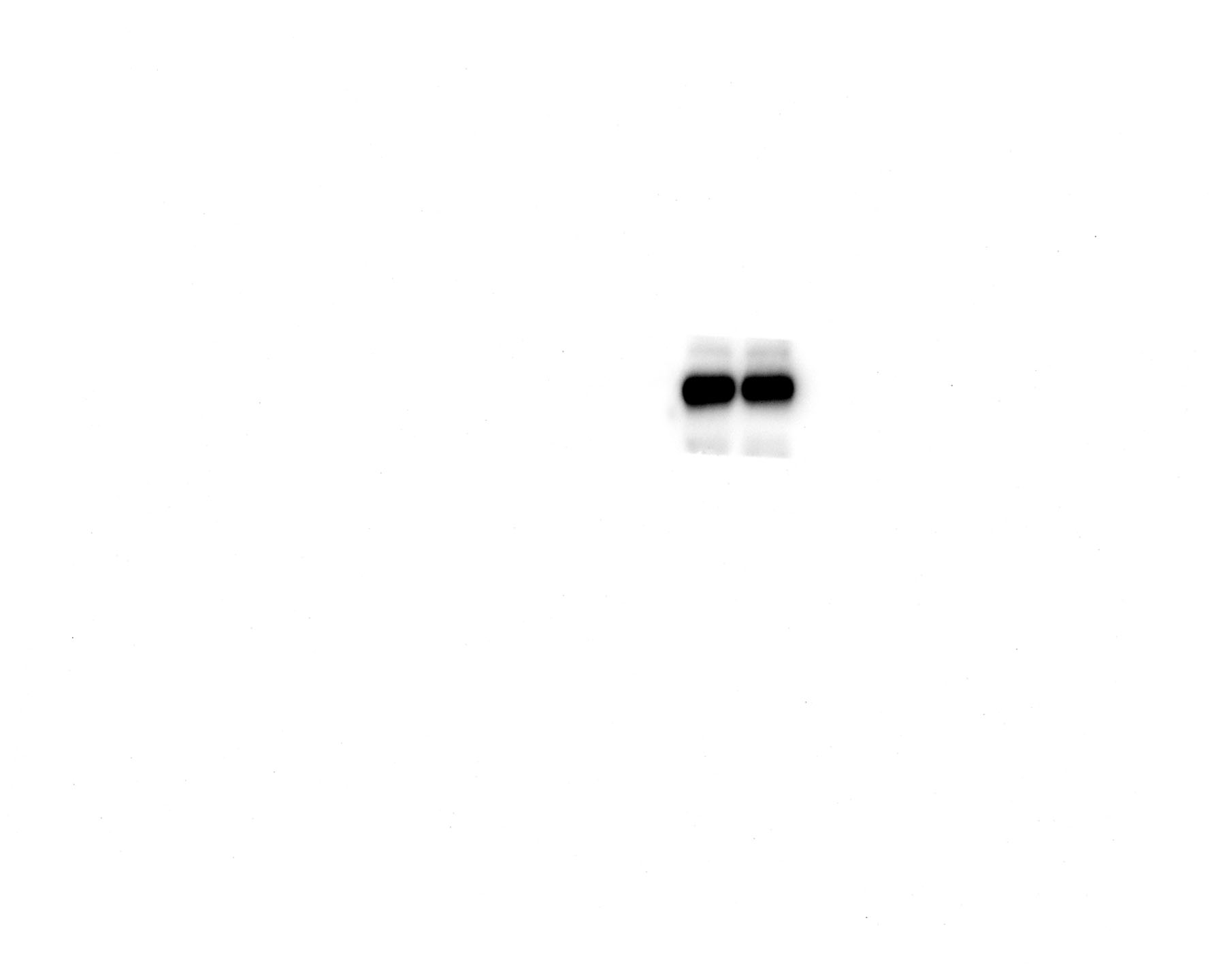


fig6b2 Input IB-flag


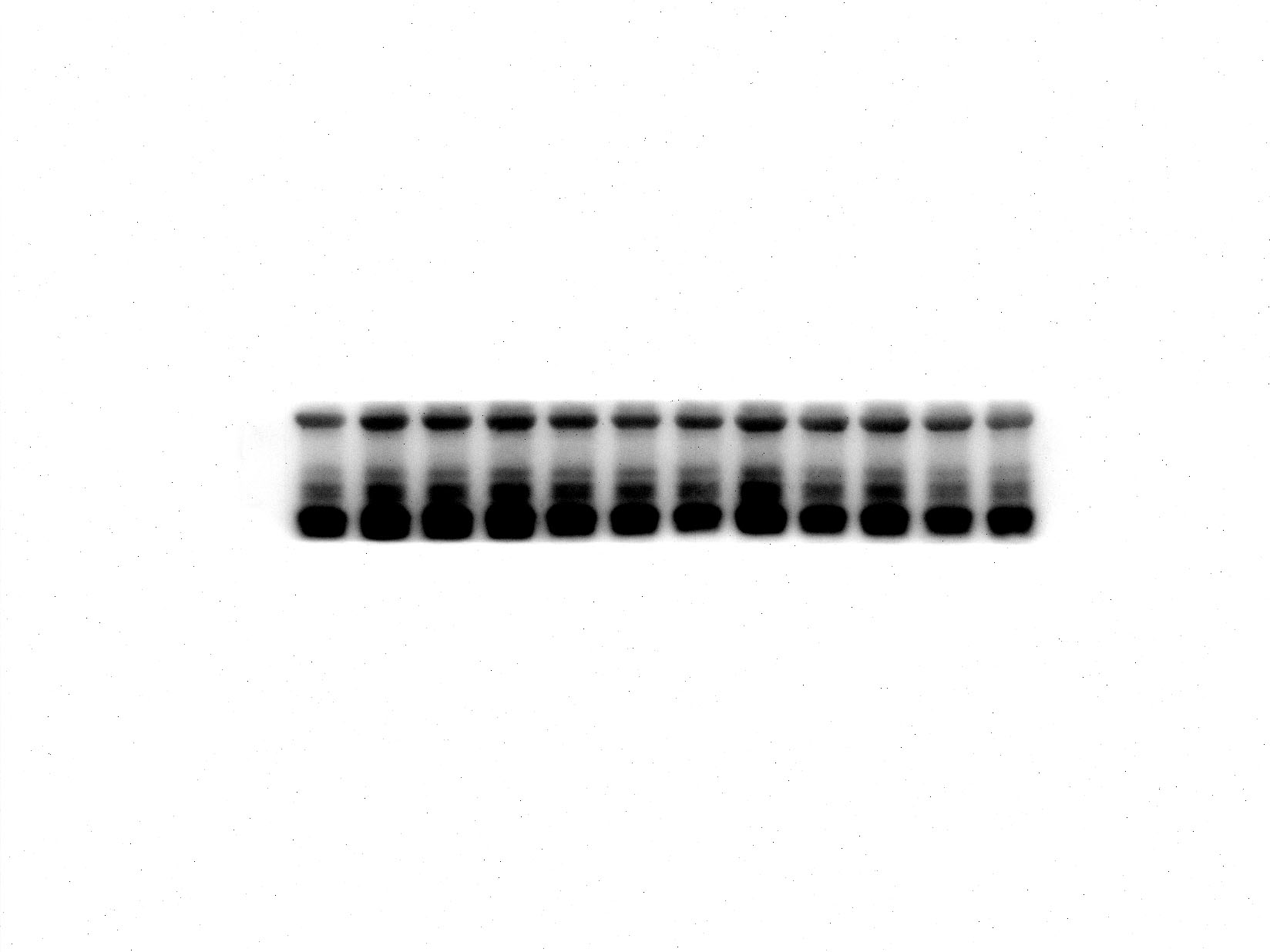


fig6b2 Input IB-Myc


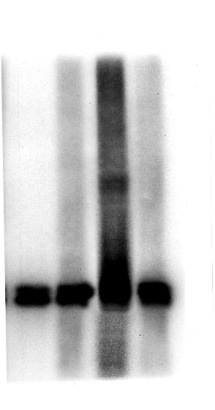


fig6b3 IP-Myc IB-HA


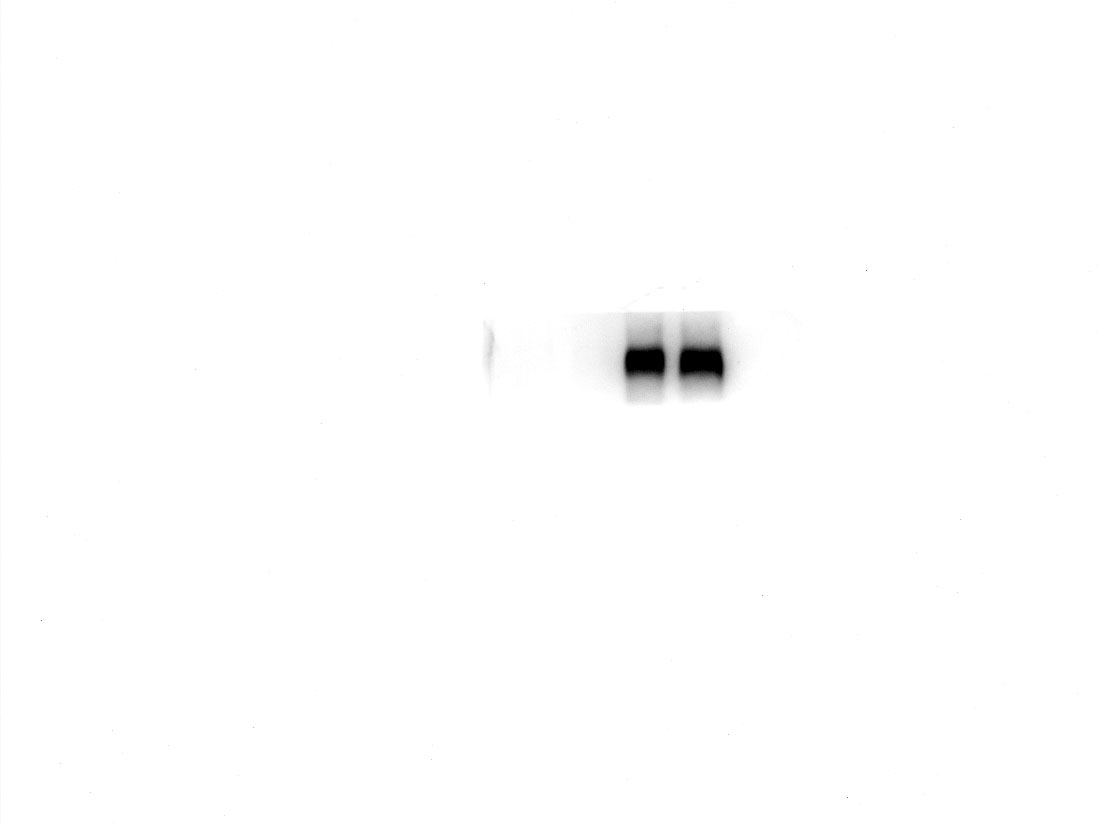


fig6b3 Input IB-flag (The image was obtained from repeated experiments and was not shown in the manuscript.)


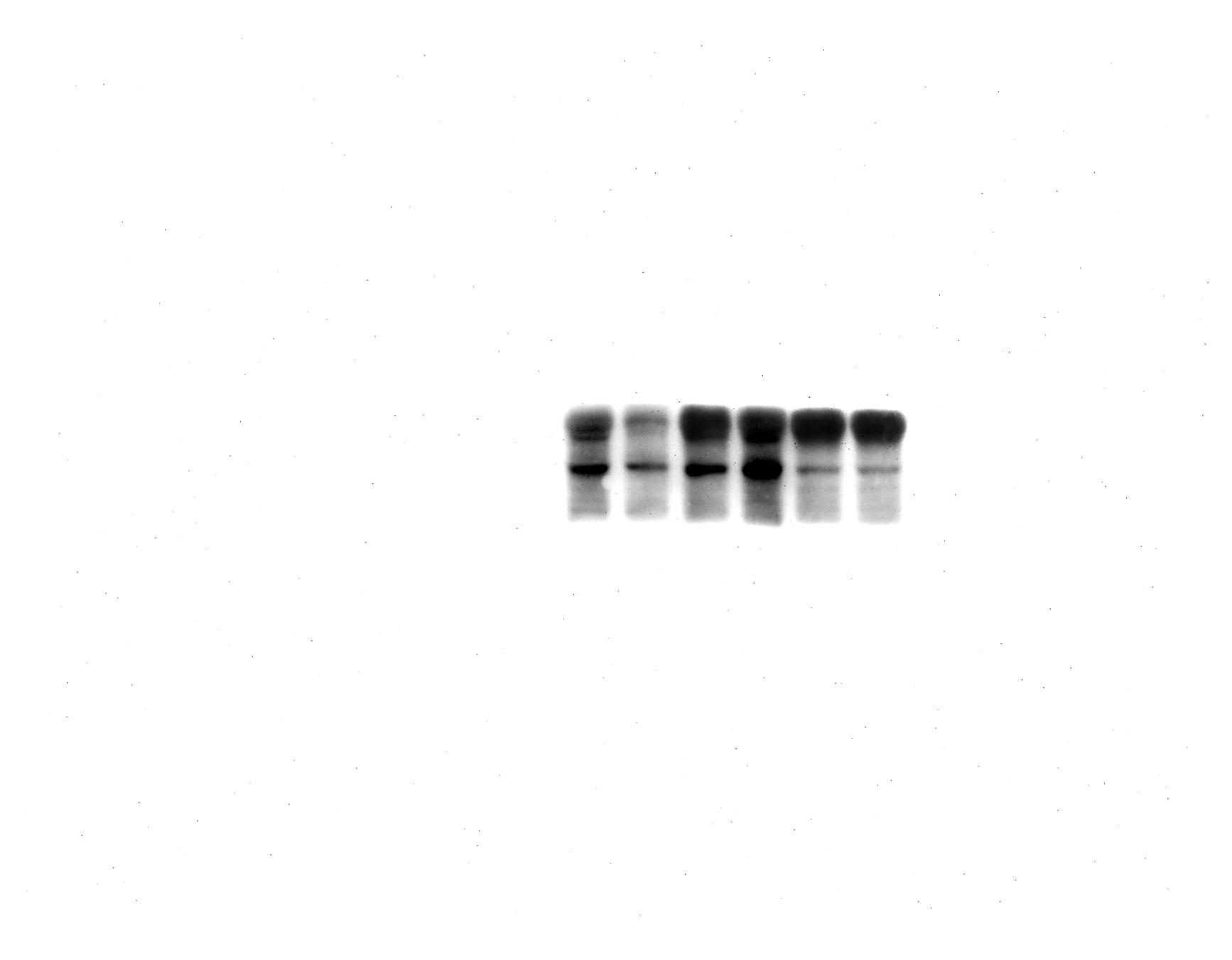


fig6b3 Input IB-Myc


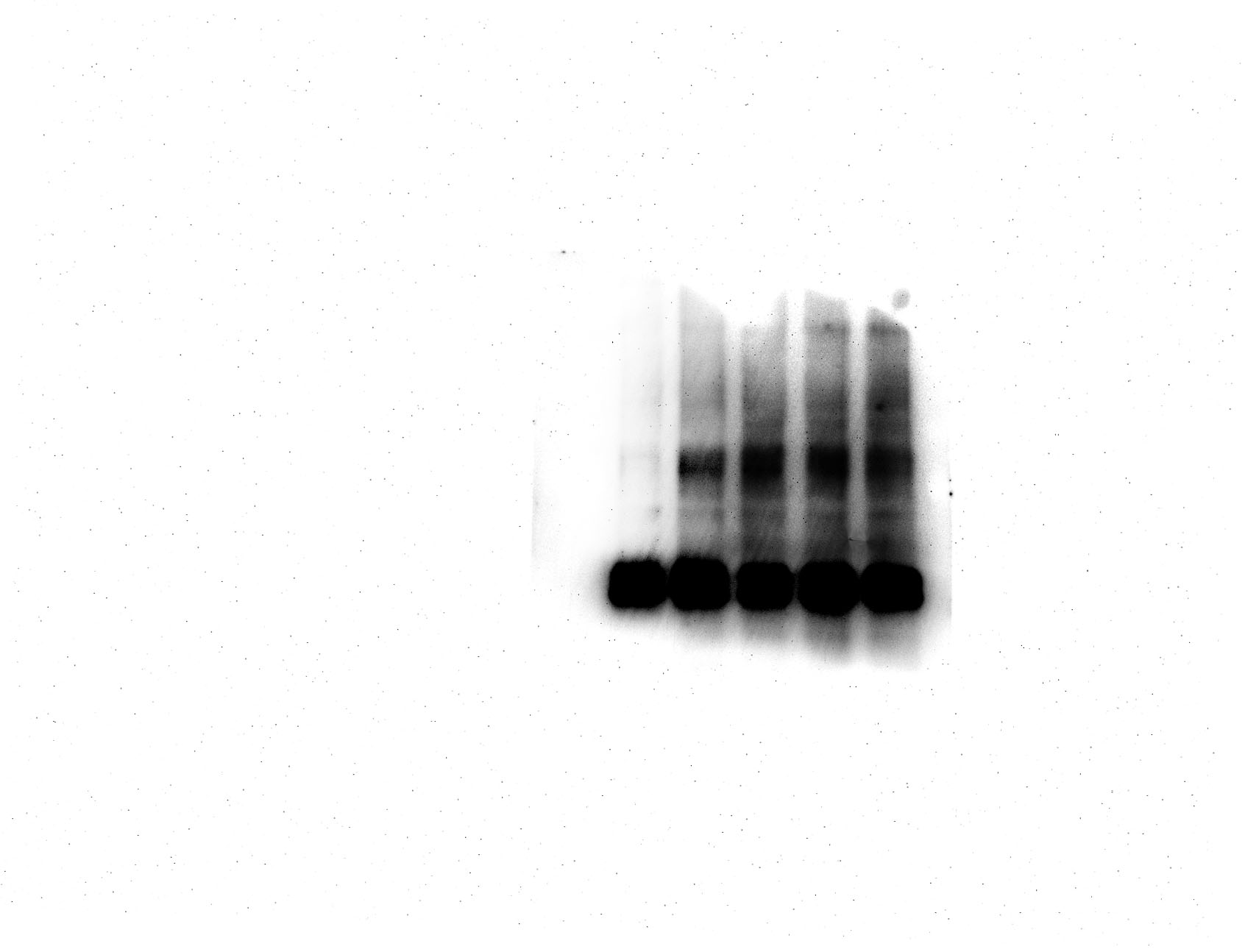


fig6c IP-Myc IB-HA


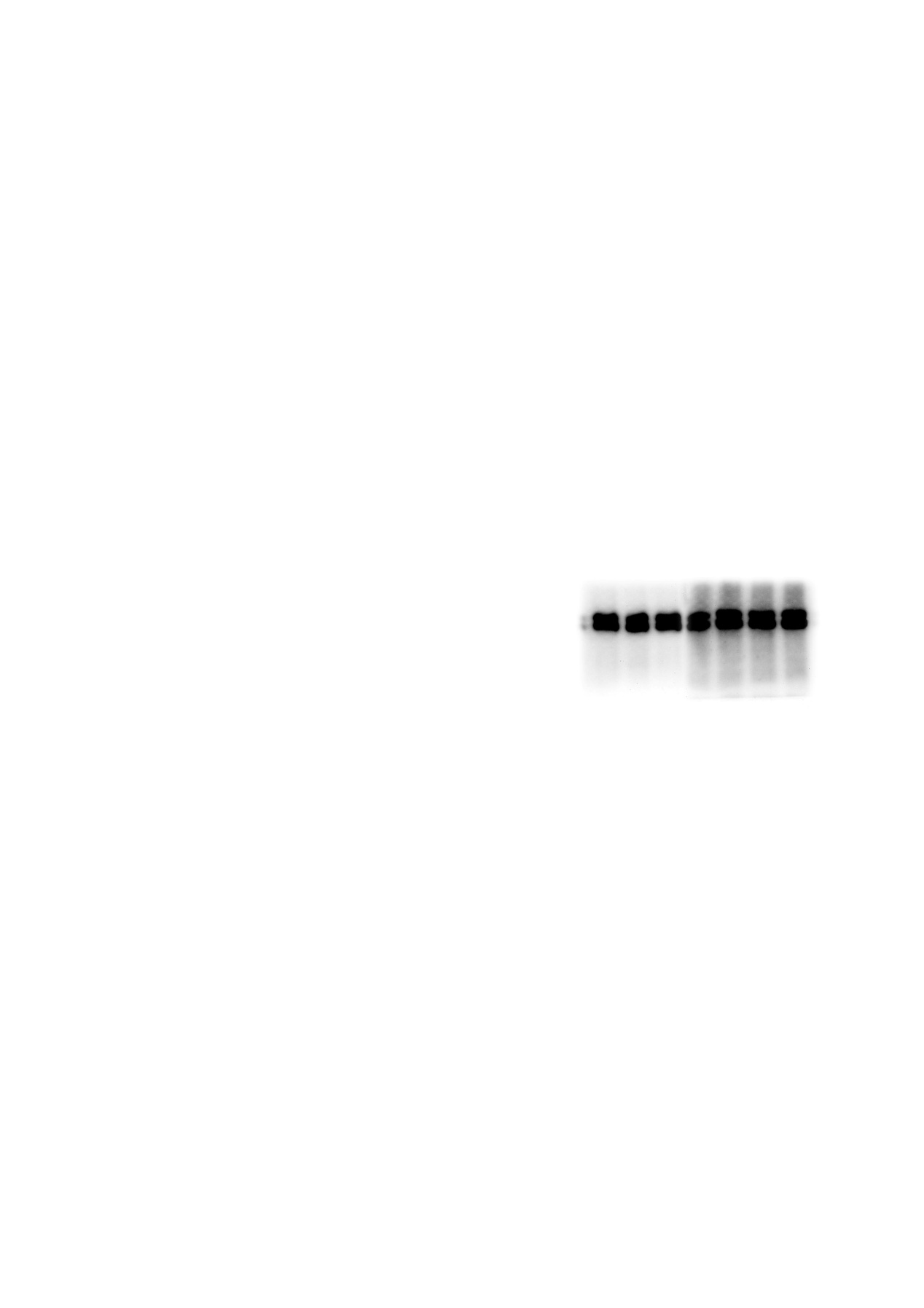


fig6c IP-Myc IB-Myc


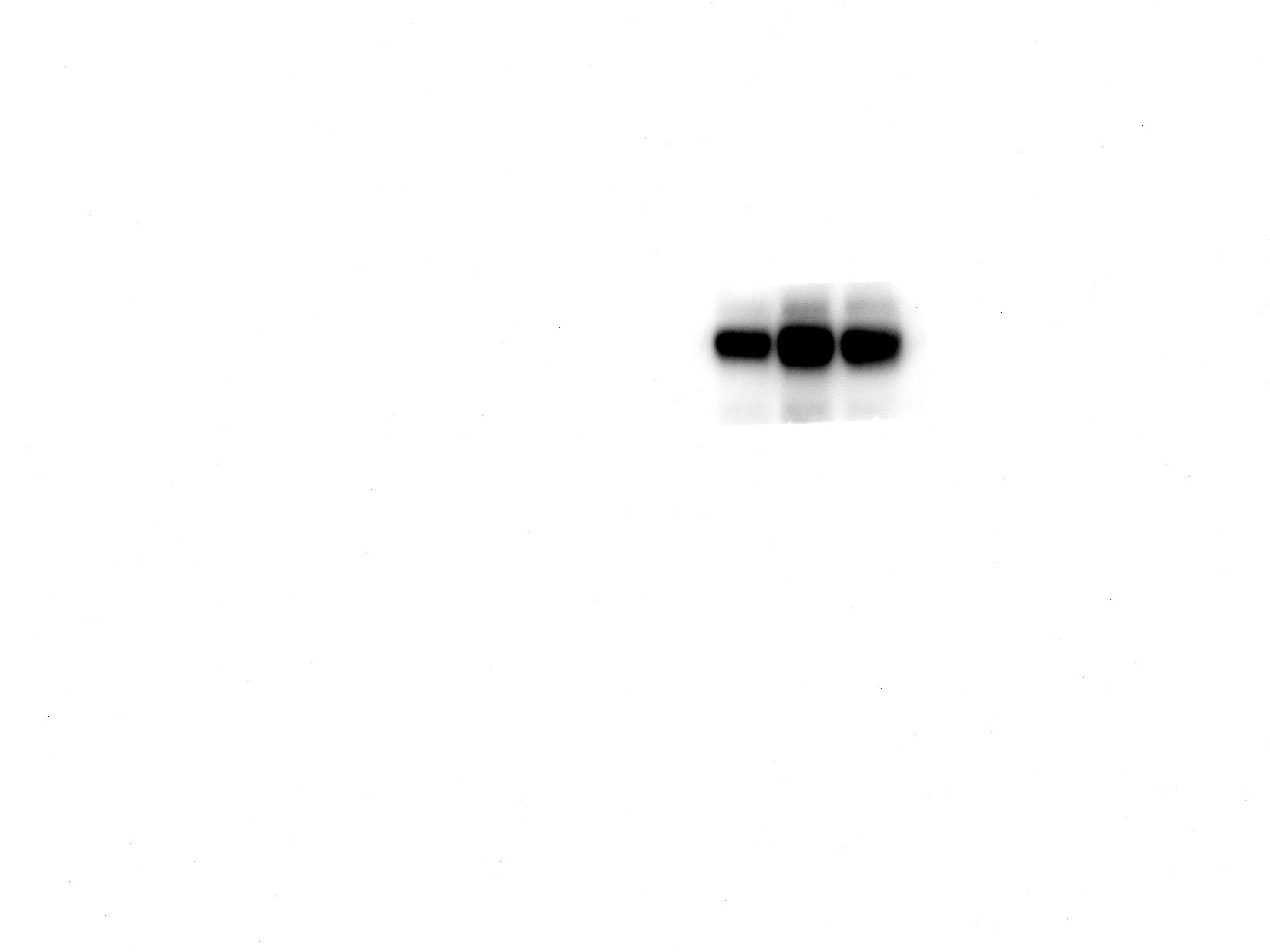


fig6c Input IB-flag


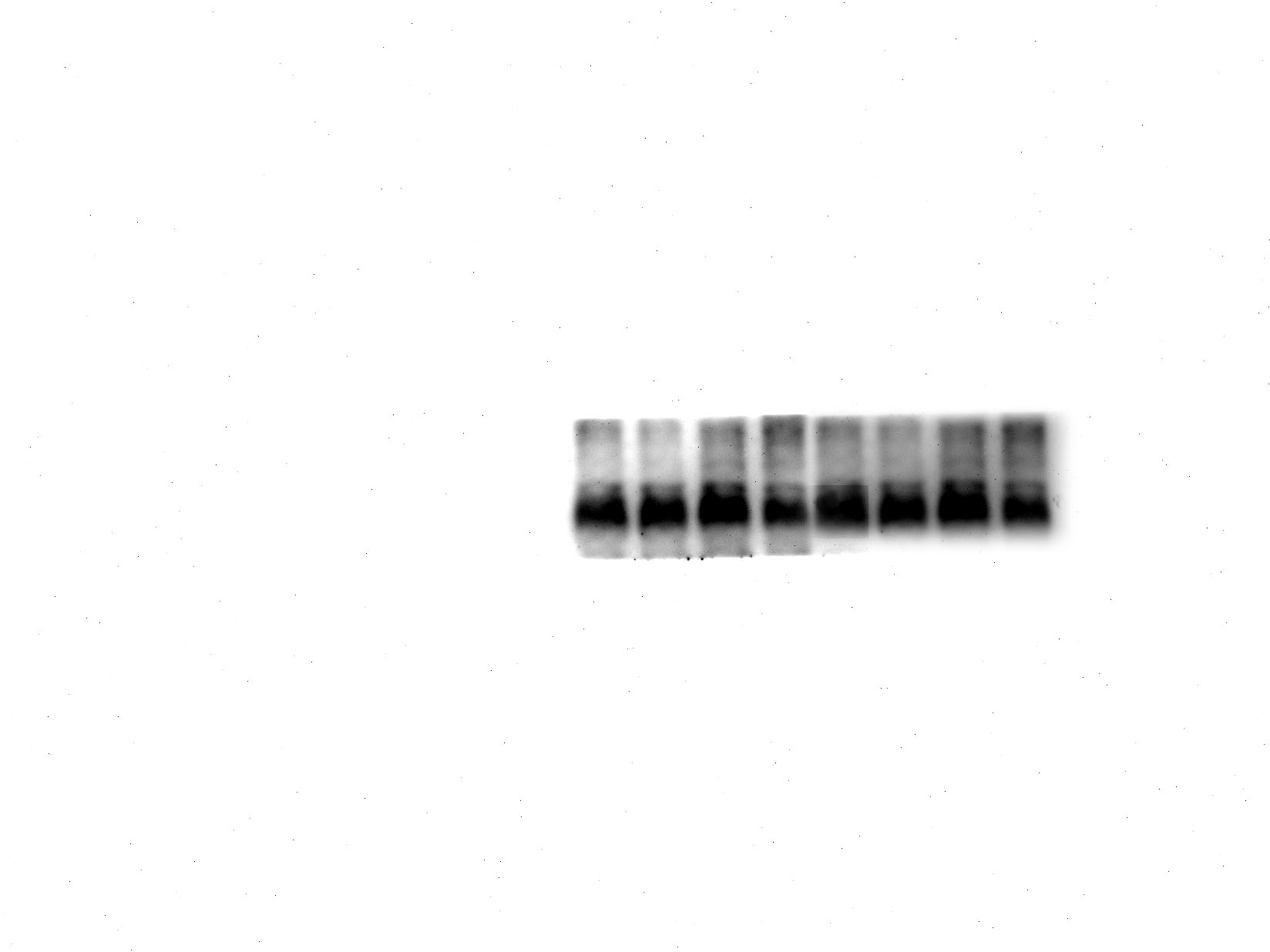


fig6c Input IB-Myc


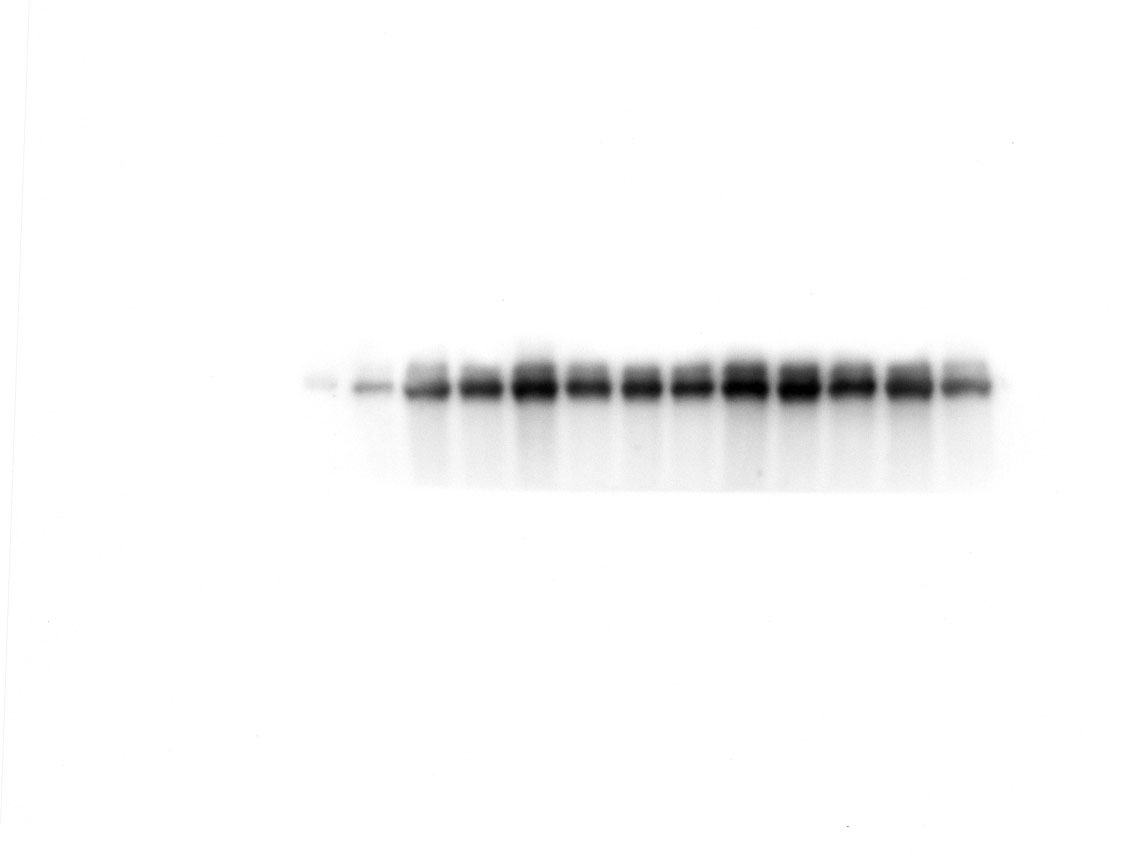


Fig7a SNO-parkin


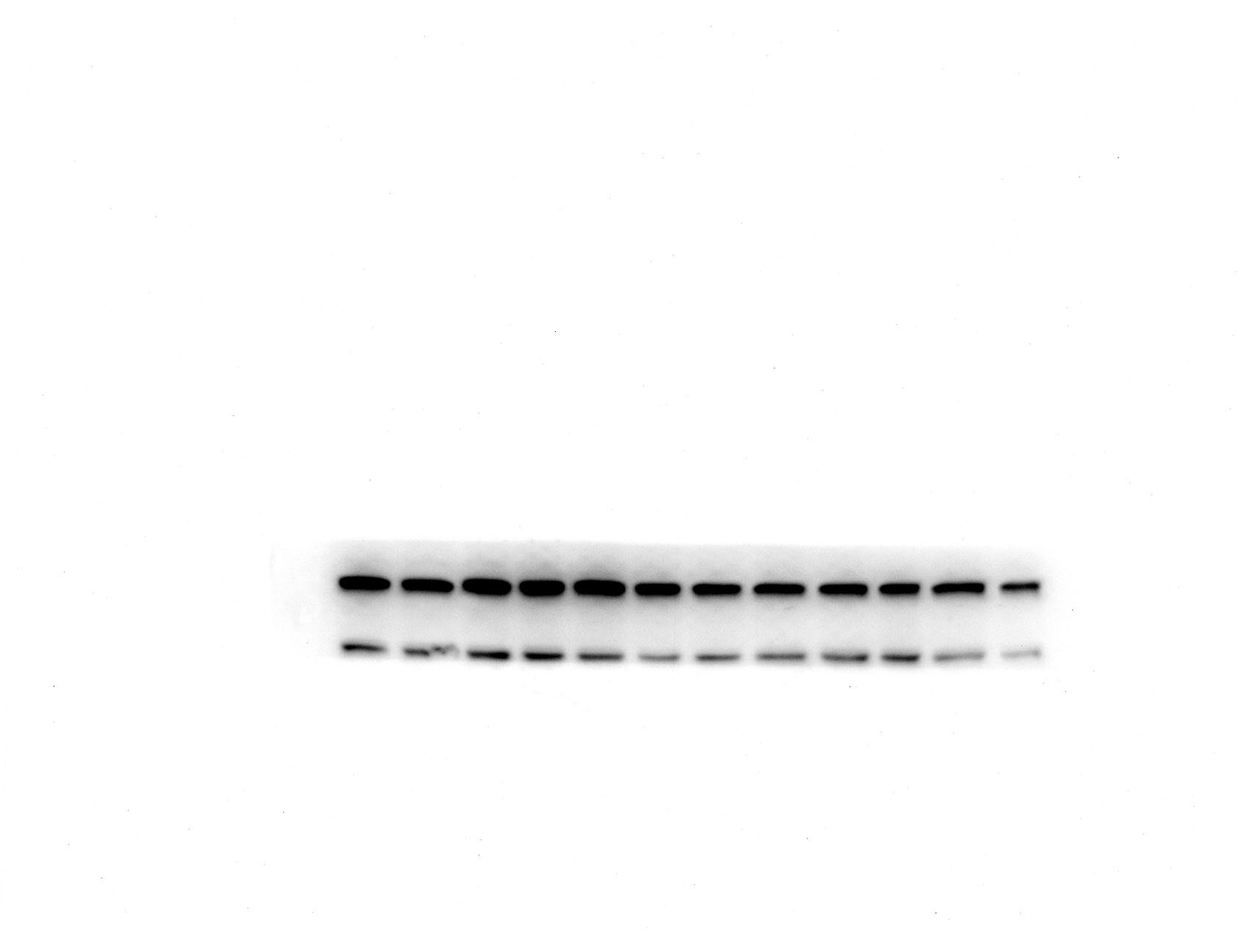


Fig7a parkin


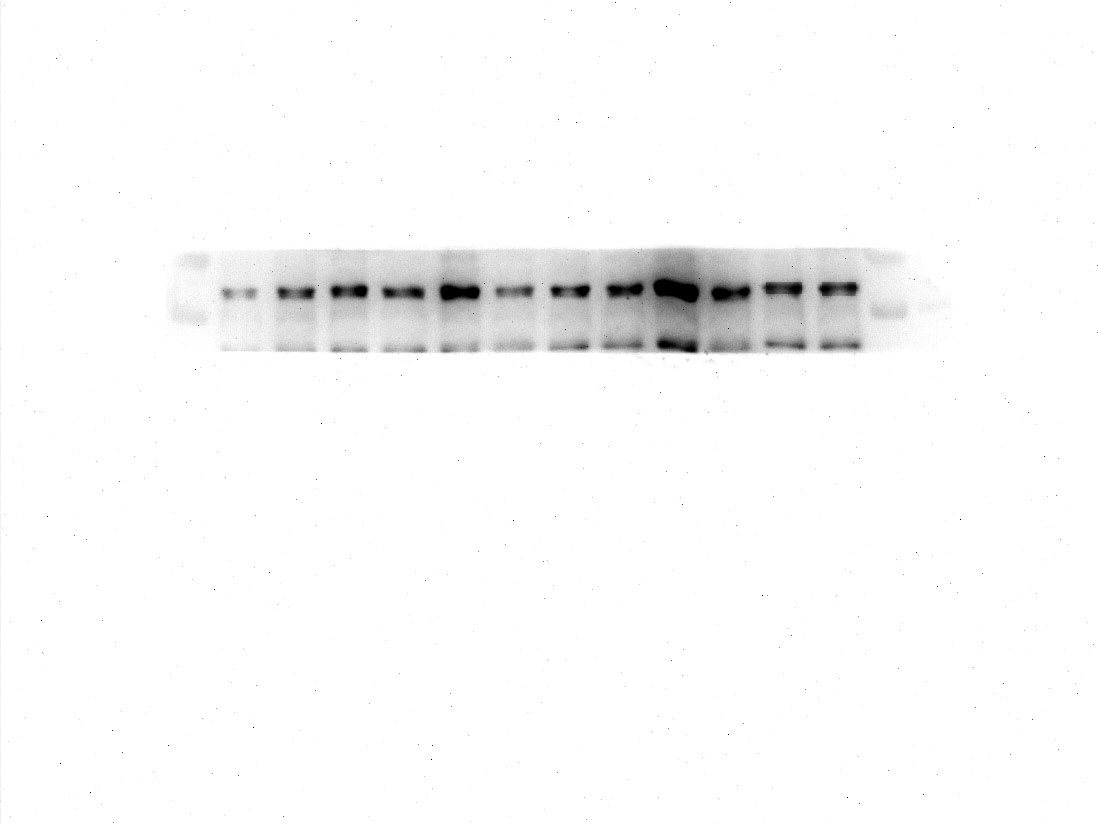


Fig7a DMT1


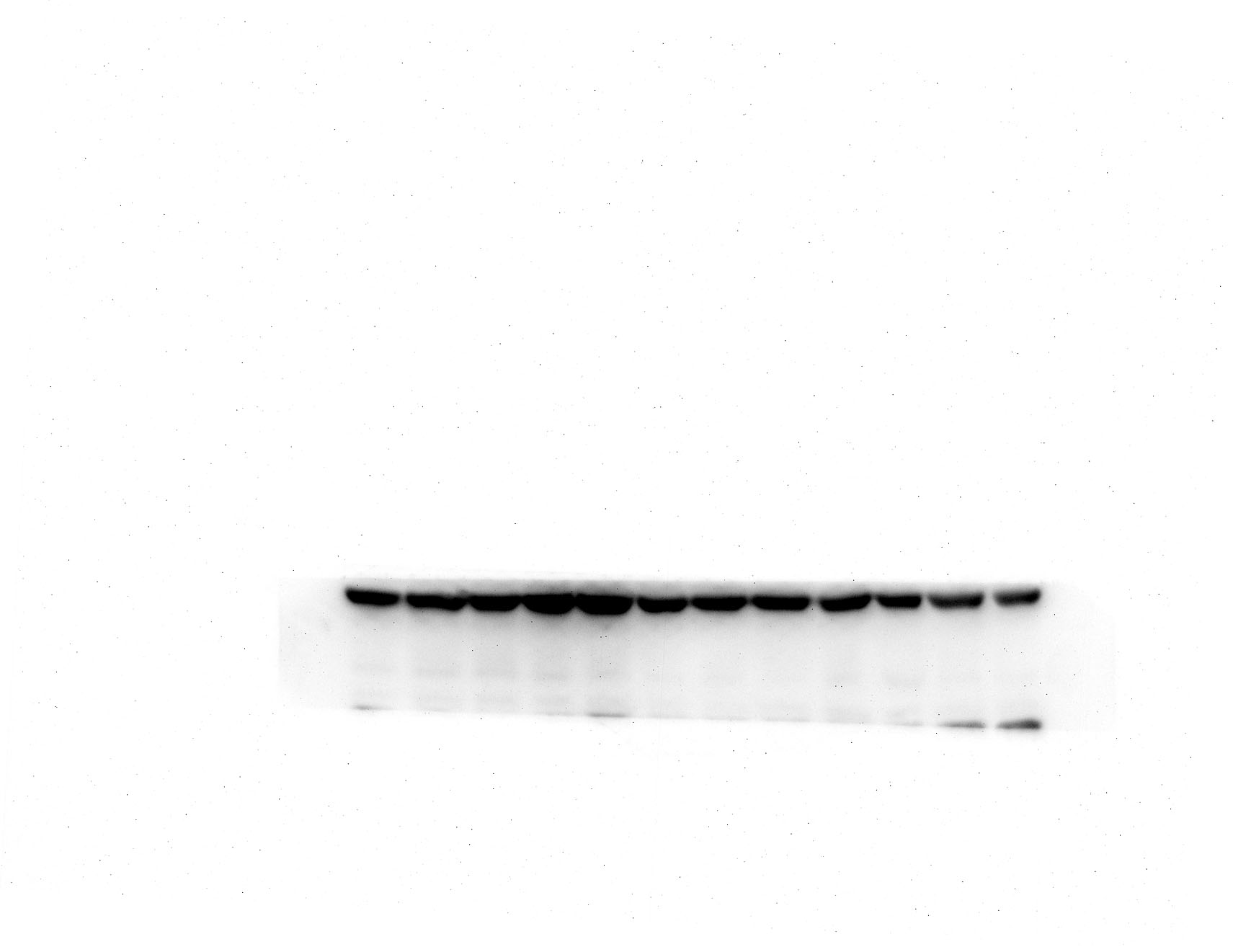


Fig7a β-actin


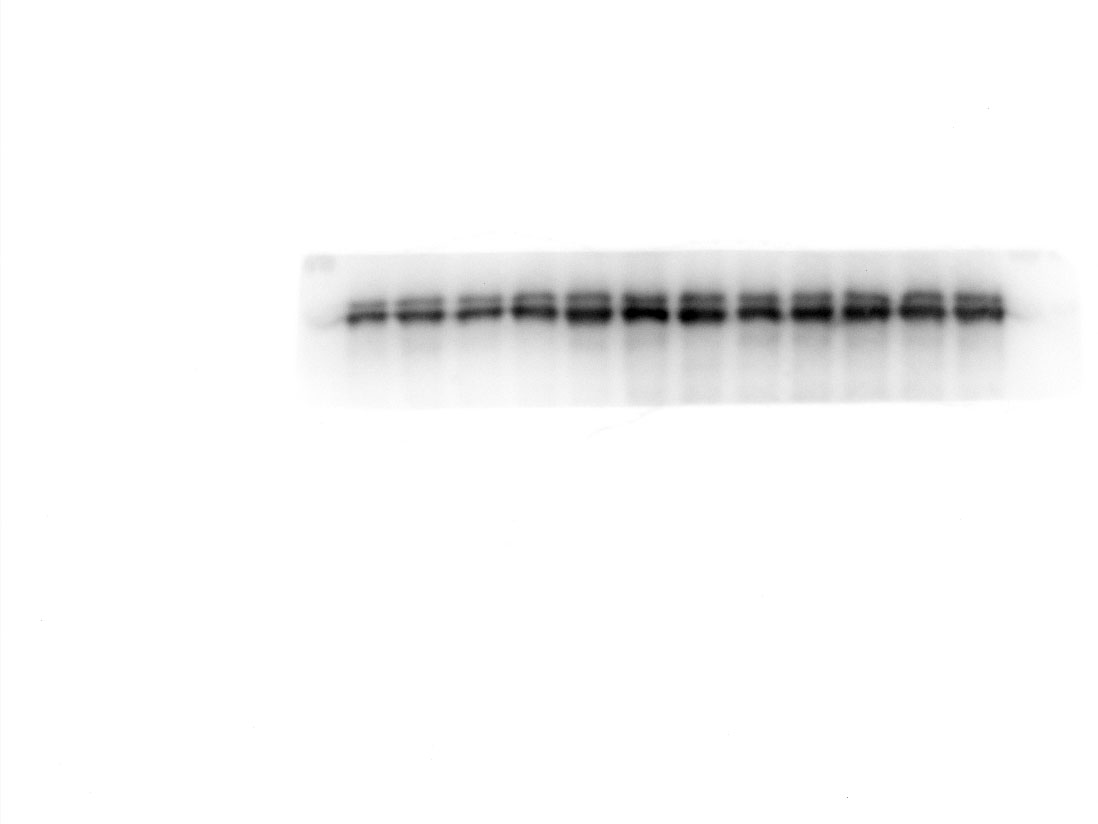


fig7d SNO-parkin


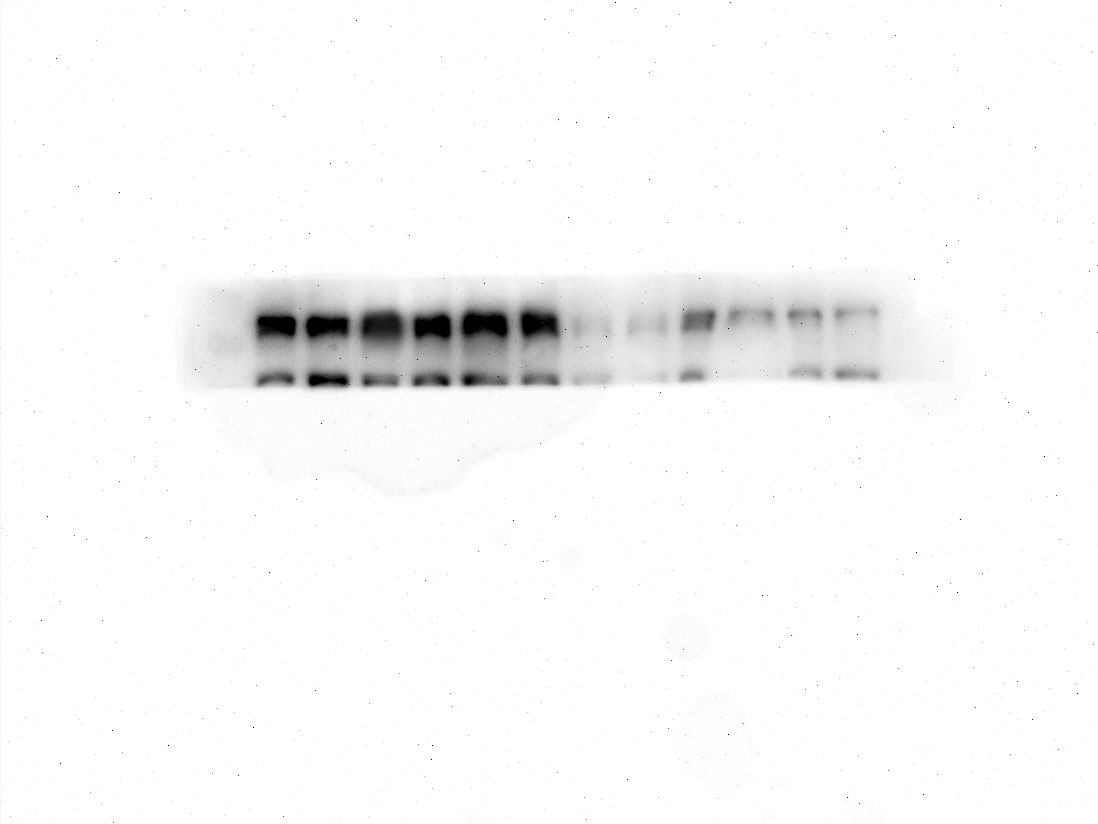


fig7d parkin


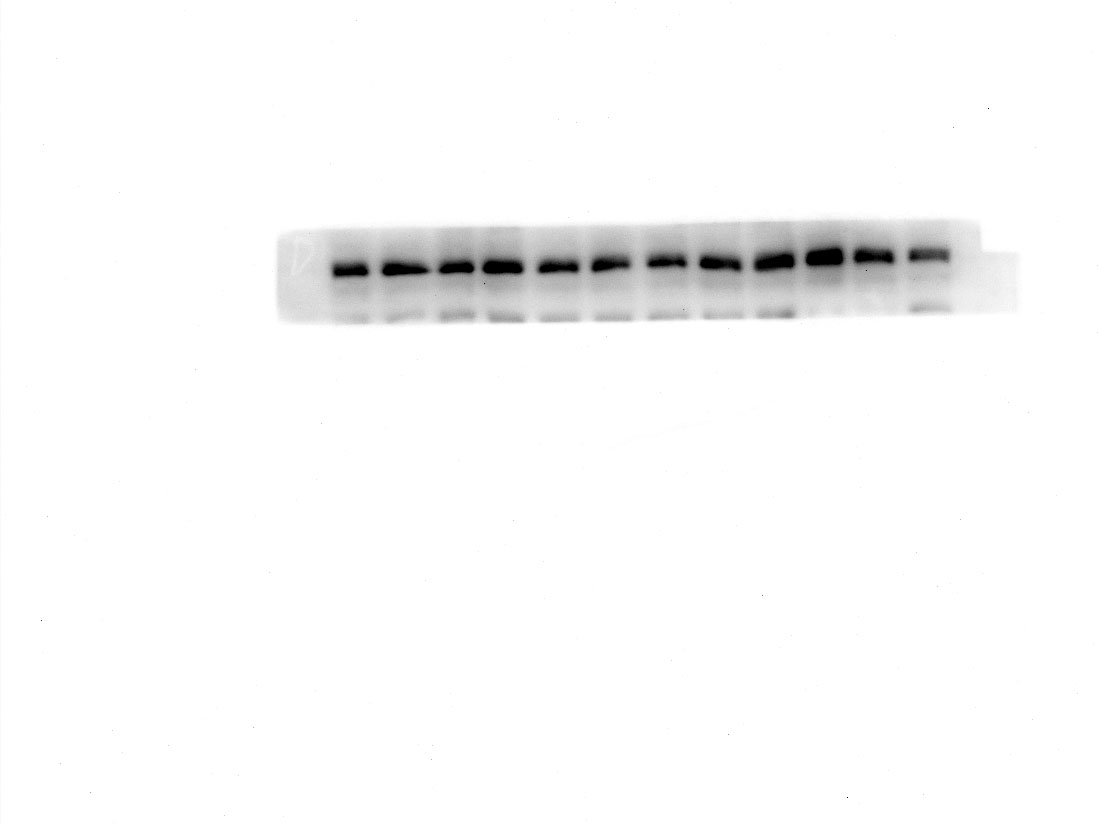


fig7d DMT1


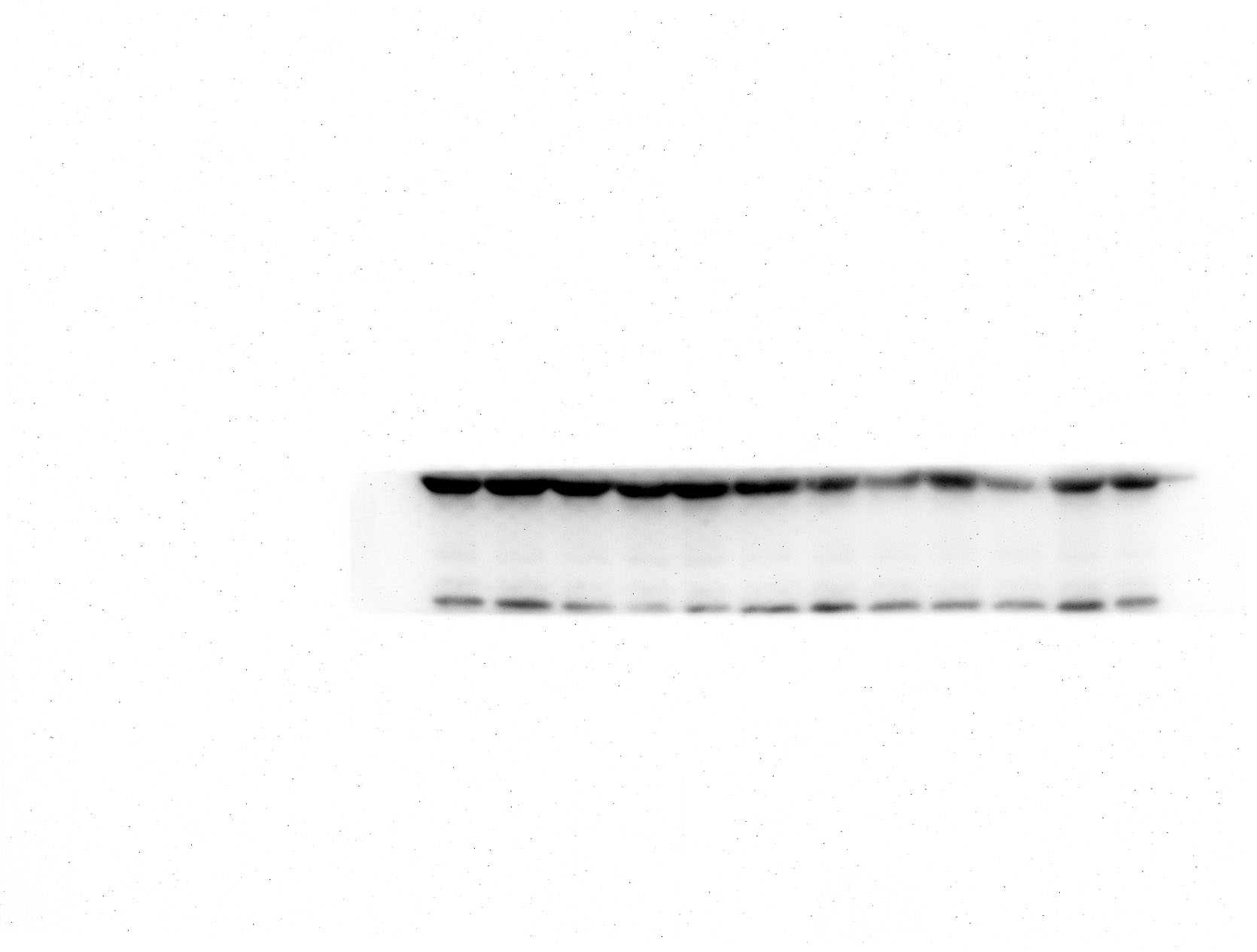


fig7d β-actin
